# Identification of glutathione metabolic genes from a dimorphic fungus *Talaromyces marneffei* and their gene expression patterns under different environmental conditions

**DOI:** 10.1038/s41598-023-40932-w

**Published:** 2023-08-24

**Authors:** Tanaporn Wangsanut, Panwarit Sukantamala, Monsicha Pongpom

**Affiliations:** Department of Microbiology, Faculty of Medicine, Chiang Mai University, Chiang Mai, Thailand

**Keywords:** Fungi, Pathogens, Fungal infection, Microbiology, Fungal genes

## Abstract

*Talaromyces marneffei* is a human fungal pathogen that causes endemic opportunistic infections, especially in Southeast Asia. The key virulence factors of *T. marneffei* are the ability to survive host-derived heat and oxidative stress, and the ability to convert morphology from environmental mold to fission yeast forms during infection. Glutathione metabolism plays an essential role in stress response and cellular development in multiple organisms. However, the role of the glutathione system in *T. marneffei* is elusive. Here, we identified the genes encoding principal enzymes associated with glutathione metabolism in *T. marneffei*, including glutathione biosynthetic enzymes (Gcs1 and Gcs2), glutathione peroxidase (Gpx1), glutathione reductase (Glr1), and a family of glutathione S-transferase (Gst). Sequence homology search revealed an extended family of the TmGst proteins, consisting of 20 TmGsts that could be divided into several classes. Expression analysis revealed that cells in conidia, mold, and yeast phases exhibited distinct expression profiles of glutathione-related genes. Also, TmGst genes were highly upregulated in response to hydrogen peroxide and xenobiotic exposure. Altogether, our findings suggest that *T. marneffei* transcriptionally regulates the glutathione genes under stress conditions in a cell-type-specific manner. This study could aid in understanding the role of glutathione in thermal-induced dimorphism and stress response.

## Introduction

Talaromycosis is caused by *Talaromyces marneffei,* an emerging opportunistic fungal pathogen that is endemic to countries in the tropical and subtropical zones of Asia, such as China, Taiwan, Hong Kong, Thailand, Laos, Vietnam, and northeastern India^1–3^. The ecosystems in these areas are usually associated with high humidity, which could promote the expansion of the fungal reservoir in the environment^4^. In endemic areas, there are approximately over 173,000 talaromycosis cases with 4900 associated deaths occurring annually^4^. *Talaromyces marneffei* primarily infects immunocompromised individuals, and infections are highly intertwined with people living in poor, rural areas where they are exposed to crops, livestock, and soils. Indeed, the risk of developing talaromycosis was 70–90% higher in farmers than in non-farmers^5^. Overall, talaromycosis is associated with high mortality and morbidity, and predisposing factors are intricately linked with weakened immune systems.

*T. marneffei* is a thermal dimorphic fungus that grows as filamentous hyphae at environmental temperatures (25 °C) and undergoes morphological switching to yeast form at human body temperature (37 °C)^6^. Conidia, the infectious asexual spores from the environment, can be inhaled into a patient’s lungs and subsequently engulfed by alveolar macrophages, where the conidia switch to the pathogenic yeast cells and cause infection^7,8^. Inside macrophages, *T. marneffei* grows as a fission yeast, but in the extracellular environment, it will appear as an arthroconidia-like yeast cell. Mechanisms that control dimorphism are still poorly understood. A number of factors involved with morphogenesis and phase transition have been partially identified^9,10^. For example, these factors are related to transcriptional regulation, G-protein signaling, and kinases^11^. Hence, more studies are urgently needed to identify key regulators that orchestrate the dimorphic switching process.

One of the key pathogenic factors is the antioxidant system. During infection, the host immune cells generate the reactive oxygen species (ROS) and reactive nitrogen species (RNS) to kill invading pathogens^12–15^. In response to the host-derived oxidative stress, *T. marneffei* activates the MAPK signaling pathway and several transcription factors to generate antioxidant proteins, which ultimately are used to neutralize the host-generated ROS/RNS^16^. These antioxidant molecules include superoxide dismutase (*sodA*), catalase-peroxidase (*cpeA*), and glutathione peroxidase (*gpx1*) that are upregulated in the pathogenic yeast form^17–19^. The *sodA* and *cpeA* were shown to be elevated early at 2 h after being phagocytosed^17,18^. Moreover, the *cpeA* deletion mutant showed decreased tolerance to hydrogen peroxide, suggesting its antioxidant role in this fungus^18^. These pioneering studies indicated that the antioxidant systems contribute significantly to the ability of this fungus to survive inside the host macrophage, and hence the success of *T. marneffei* in pathogenesis.

Glutathione (GSH, L-γ-glutamylcysteinylglycine) is a crucial metabolite in eukaryotes and plays a major role in protecting cells against oxidative damage^20^. Glutathione directly scavenges diverse oxidants such as superoxide anions, hydroxyl radicals, carbon radicals, and nitric oxide^20^. In microbial pathogens, glutathione can function as a signaling molecule to modulate virulence pathways^21–23^. In yeast population, glutathione is secreted and accumulated extracellularly to combat harmful chemicals arising from unhabitable high temperature^24^. In addition, glutathione is a cofactor for various antioxidant enzymes such as glutathione peroxidase and glutathione S-transferase^25,26^. There are two states of glutathione in the cells: reduced glutathione (GSH) and oxidized glutathione disulfide (GSSG). Under non-stress conditions, GSH is a major antioxidant while GSSG is accumulated when cells are exposed to increased levels of oxidative stress. In general, increased ratios of GSSG to GSH are indicative of oxidative stress. Thus, cells tightly maintain levels of reduced glutathione through the balance of its synthesis and reduction. The glutathione homeostasis system mainly contains five enzymes, γ -glutamylcysteine synthetase and glutathione synthetase involved in glutathione biosynthesis, glutathione peroxidase and glutathione reductase involved in recycling of the GSH, and glutathione S-transferase involved in detoxification of several stressors^20,25,27^. Notably, the glutathione S-transferase proteins represent an extended family of multifunctional enzymes that are involved in diverse pathways such as oxidative stress response, and detoxification of various substrates^28^. For instance, Gsts detoxify xenobiotic substrates by conjugating GSH with certain xenobiotic compounds, thus enhancing the excretion of the xenobiotics from the cells. Despite the pivotal role in the cells, the function of the glutathione system in cellular survival and dimorphism has not been fully characterized in *T. marneffei*.

Here, we reported the entire glutathione system in *T. marneffei*. Furthermore, the involvement of the glutathione system in different cell types of *T. marneffei* and under oxidative stress conditions were explored. We characterized the genes encoding for glutathione biosynthetic enzymes, glutathione reductase (*glr*), glutathione peroxidase (*gpx*) and glutathione S-transferases (*gsts*). The 20 *gst* genes from *T. marneffei* were discovered by conducting a genome-wide annotation search, and then classified by performing phylogenetic tree analysis. We assessed the expression patterns and transcript abundance of these genes in relation to conidia, mold, and yeast phases. By analyzing the gene expression profile from two different *T. marneffei* strains, we found that several glutathione-related genes exhibit phase-specific expression patterns. The TmGst-encoding genes were strongly induced upon exposure to oxidative and xenobiotic stressors. This study provides the first transcript-level insights into the glutathione gene family of *T. marneffei* that might contribute to morphological switching and adaptation to oxidative stress during host infection.

## Results

### Sequence analyses

**Figure 1:**
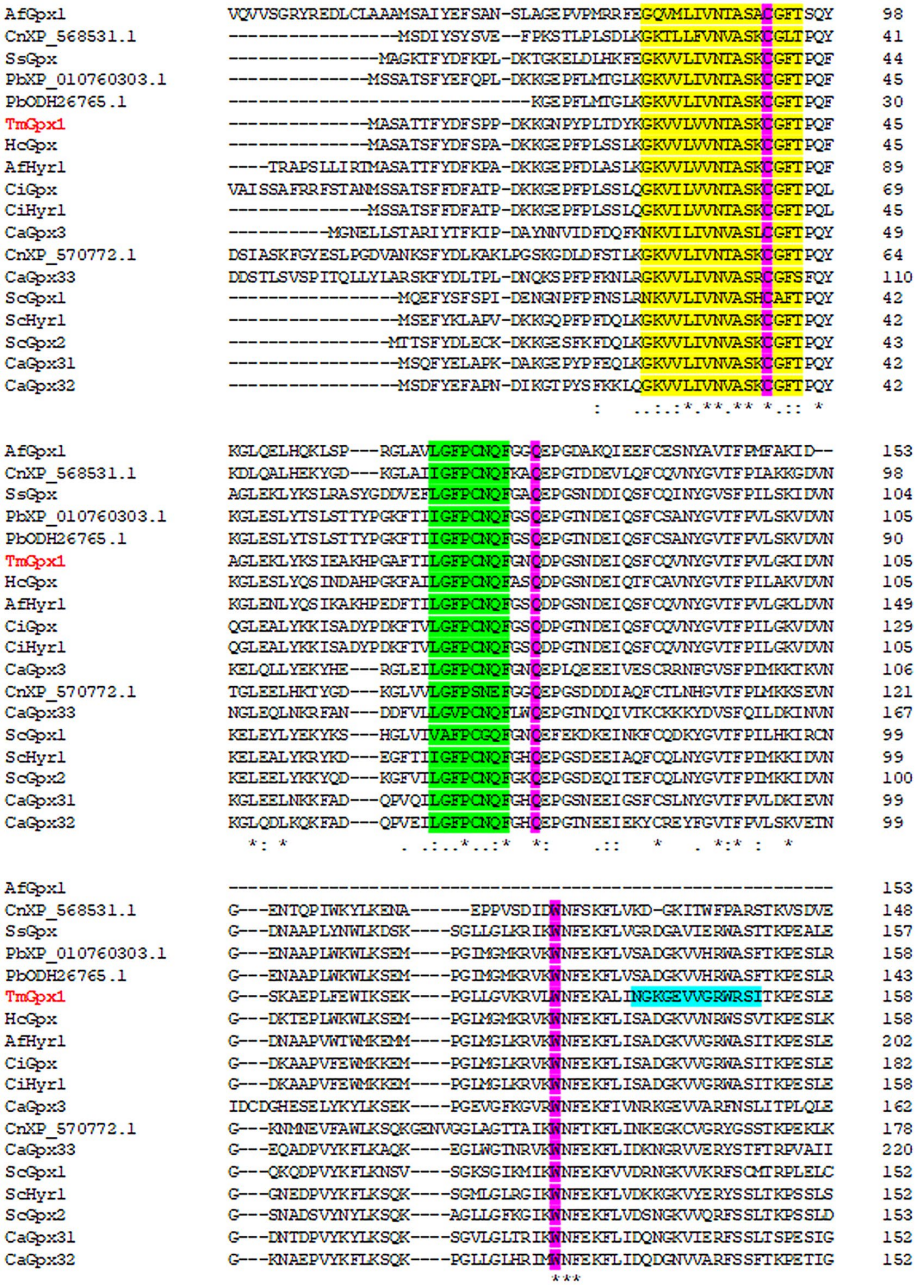
TmGpx1 (TmHyr1) shows high homology with other fungal glutathione peroxidases and contained the conserved catalytic triad CQW. Protein sequences were obtained from BLAST analysis. Protein sequence alignment was performed using Clustal Omega. Functional domains were obtained using the web-based analysis tool ScanProsite. Yellow highlight = Active site (PS00460); Green highlight = Signature 2 (PS00763); Blue highlight = lipocalin signature (PS00213); Magenta highlight = Residues constituting the catalytic triad CQW. The symbols below the alignment indicate the conservation: asterisk (*) indicates a single fully conserved amino acid, colon (:) indicates amino acid groups with strongly similar properties, and dot (·) indicates groups with weakly similar properties. Fungal species are abbreviated as followed; Af: *Aspergillus fumigatus*, Cn: *Cryptococcus neoformans*, Ss: *Sporothrix schenckii*, Pb: *Paracoccidioides brasiliensis*, Tm: *Talaromyces marneffei*, Hc: *Histoplasma capsulatum*, Ci: *Coccidioides immitis*, Sc: *Saccharomyces cerevisiae*, Ca: *Candida albicans*.

We searched *T. marneffei* annotated genome and collected putative genes encoding for glutathione metabolic enzymes (Tables 1 and 2). All glutathione-related genes are conserved in this fungus. For glutathione peroxidase, *T. marneffei* possesses only one homolog, which is closely related to the glutathione peroxidase Hyr1 (Gpx3) protein from *Saccharomyces cerevisiae*. The gene has been named *gpx1*^29^. The amino acid sequences of glutathione peroxidase from *T. marneffei* were used in homology searches against other common human fungal pathogens (Fig. 1). Alignment of amino acid sequences shows that TmGpx1 shares a high identity with *Aspergillus fumigatus* glutathione peroxidase Hyr1 (77%), *Histoplasma capsulatum* glutathione peroxidase (73%), *Coccidioides immitis* glutathione peroxidase Hyr1 (73%), and *Paracoccidioides brasiliensis* Pb18 glutathione peroxidase (67%). As opposed to high conservation among pathogenic fungi, TmGpx1 shares only 40% identity with the human Gpx7 and Gpx8 proteins. The residues selenium-cysteine (U) or cysteine (C) at the active site, together with glutamine (Q) and tryptophan (W), forms the signature “catalytic triad” of the glutathione peroxidase family^30^. The residues constituting this catalytic triad CQW are conserved in all compared sequences. The signature patterns around the triad are also highly conserved. The first conserved region contains a consensus sequence for the active site (GKVVLVVNTASKCGFT). The second conserved region is LGFPCNQF, named glutathione peroxidase signature 2. The third region was found only in TmGpx1, called a lipocalin sequence signature (NGKGEVVGRWRSI) suggesting that these sequences might have specific functions in *T. marneffei*. Altogether, the TmGpx1 protein contains both highly conserved regions as well as *T. marneffei*- specific signature sequences.

**Table 1:** Glutathione gene homologs in *T. marneffei.*

Glutathione enzyme	*T. marneffei* homolog^a^	*S. cerevisiae* homolog^b^	E-value	Identity (%)^c^
γ-glutamylcysteine synthetase (Glutamate cysteine ligase)	Glutamate cysteine ligase Gcs1 (PMAA_019080)	Glutamate cysteine ligase (NP_012434.1)	5e−153	40.57
Glutathione synthetase	Glutathione synthetase (PMAA_084690) Glutathione synthetase (PMAA_030280)	Glutathione synthetase (NP_014593.1)	5e−57 2e−52	36.96 28.19
Glutathione reductase	Glutathione oxidoreductase (PMAA_066180)	Glutathione-disulfide reductase (NP_015234.1)	2e−150	51.02
Glutathione peroxidase	Glutathione peroxidase *HYR1* (PMAA_007230)	*Peroxiredoxin HYR1*, *GPX3* (NP_012303.1) *Glutathione peroxidase GPX2* (NP_009803.3) *Glutathione peroxidase GPX1* (NP_012899.3)	2e−65 5e−64 3e−41	55.76 56.96 46.26

^a^*T. marneffei* strain ATCC18224.

^b^*S. cerevisiae* strain s288c.

^c^Identify compared to protein from *S. cerevisiae.*

**Table 2:** Characteristics of glutathione S-transferase genes identified in *T. marneffei.*

Type	Name	Gene ID	Localization^a^	Protein (aa)	Uniprot	pI^b^	Mw (Da)^b^
	TmGst1	PMAA_047710	mito	279	B6QP98	5.99	31983.65
	TmGst2	PMAA_089520	cyto	239	B6QEK7	6.61	27406.30
	TmGst3	PMAA_023710	cyto	253	B6Q5T5	6.42	28717.42
	TmGst4	PMAA_098360	mito	164	B6QIQ1	5.32	18145.74
	TmGst5	PMAA_075870	cyto	218	B6QBZ6	5.87	24392.23
	TmGst6	PMAA_048780	nucl	332	B6QPK6	6.73	37961.57
	TmGst7	PMAA_077030	cysk	232	B6QCV8	5.95	25834.69
Theta	TmGst8	PMAA_063970	mito	218	B6QAM3	5.69	24926.68
Theta	TmGst9	PMAA_076200	cyto	207	B6QC32	7.01	23362.87
	TmGst10	PMAA_064630	cyto	226	B6QB08	5.84	25339.51
	TmGst11	PMAA_091900	mito	223	B6QK31	6.34	25394.11
SAM35	TmGst12	PMAA_067880	nucl	311	B6QCN6	6.56	35186.79
EFB1	TmGst13	PMAA_012080	cyto	406	B6QVD1	6.17	45912.49
	TmGst14	PMAA_018660	cyto	238	B6Q2K1	5.30	27136.53
	TmGst15	PMAA_031260	cyto	237	B6Q4S3	5.73	26235.14
MAPEG	TmGst16	PMAA_070710	extr	191	B6Q931	9.73	21141.87
Kappa	TmGst17	PMAA_059540	pero	233	B6QM48	6.51	26655.73
	TmGst18	PMAA_041810	nucl	265	B6QQP3	6.71	30195.35
	TmGst19	PMAA_016830	cyto	208	B6Q801	7.80	23604.88
	TmGst20	PMAA_079200	mito	249	B6QE23	7.02	28047.90

^a^Localization predicted by WoLF PSORT, *mito* mitochondria, *cyto* cytoplasm, *nucl* nucleus, *cysk* cytoskeleton, *extr* extracellular space, *pero* peroxisome.

^b^pI and Molecular Weight (Mw) were predicted using the Compute pI/Mw tool (https://web.expasy.org/compute_pi/).

**Figure 2:**
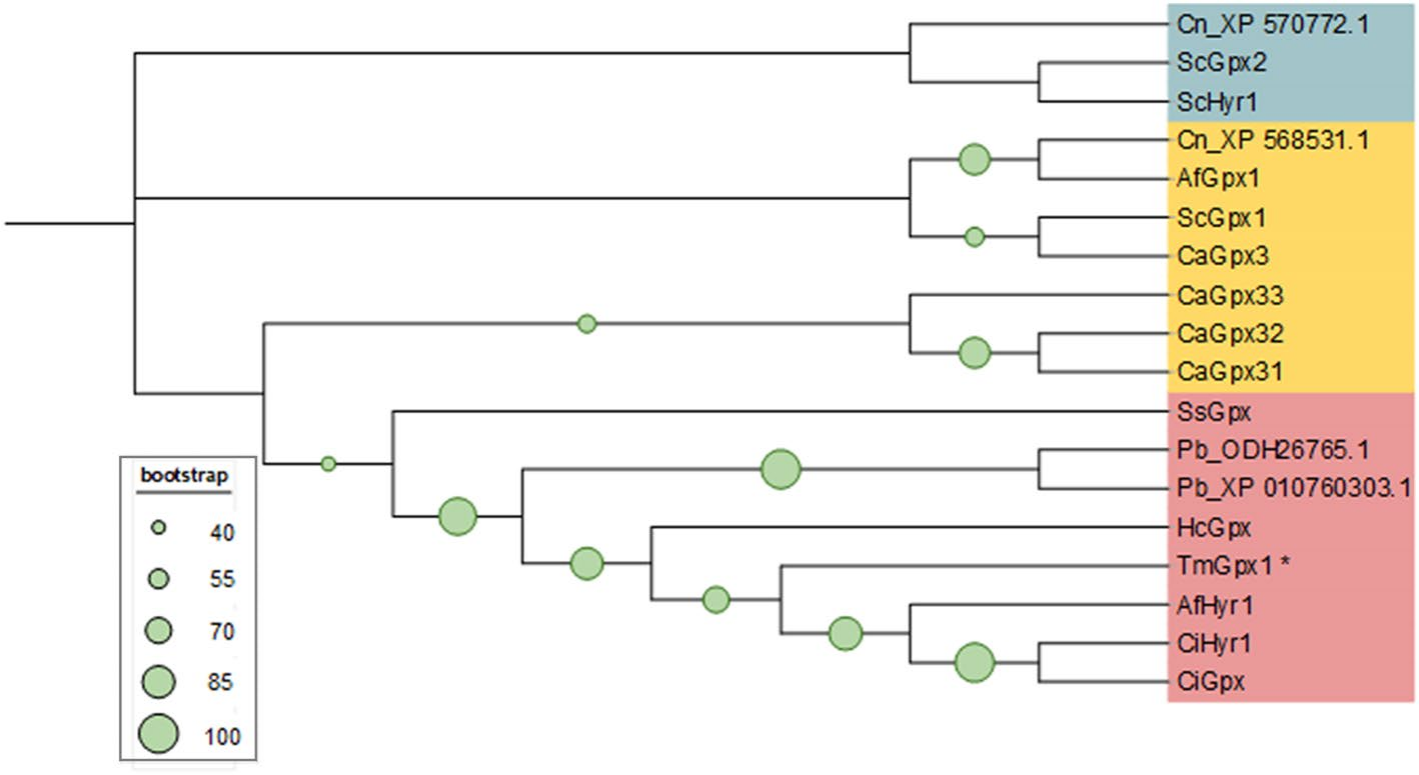
Phylogenetic tree analysis of glutathione peroxidase in common pathogenic fungi. Amino acid sequences of these proteins were obtained as described in Fig. 1. Neighbor-joining phylogenic tree of the Gpx1 family was constructed using MEGA software with Bootstrap value equal to 1000. Green circle at nodes of the phylogenetic tree represent bootstrap values, and only the values higher than 40 are displayed. Three clades of glutathione proteins are shaded in different colors. The asterisk indicates glutathione peroxidase from *T. marneffei*. The accession numbers of all proteins are listed in supplemental data S1. Fungal species are abbreviated as follows; Af: *Aspergillus fumigatus*, Cn: *Cryptococcus neoformans*, Ss: *Sporothrix schenckii*, Pb: *Paracoccidioides brasiliensis*, Tm: *Talaromyces marneffei*, Hc: *Histoplasma capsulatum*, Ci: *Coccidioides immitis*, Sc: *Saccharomyces cerevisiae*, Ca: *Candida albicans*.

To understand the evolutionary relationship among fungal Gpx proteins, we constructed a phylogenetic tree using MEGA 11 programs. Based on phylogenetic tree analysis, TmGpx1 is the most closely related to Hyr1 from *A. fumigatus* (Fig. 2). In addition, TmGpx1 is more closely related to the filamentous fungus *Aspergillus* and other thermal dimorphic fungi than yeasts such as *S. cerevisiae*, *Candida albicans*, and *Cryptococcus neoformans*.

### Identification of *T. marneffei* Gst protein family

**Figure 3:**
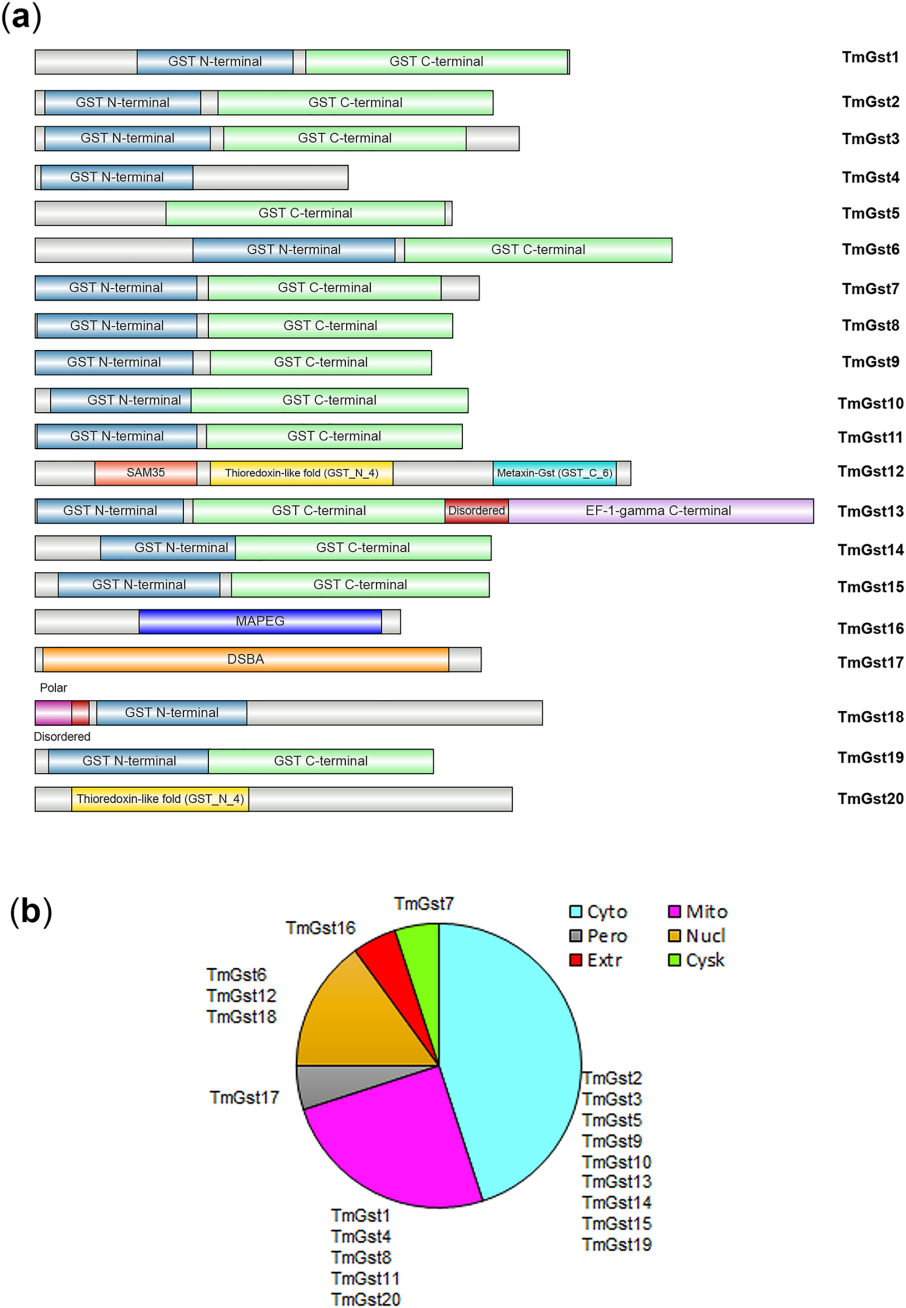
Characteristics of 20 Gsts from *T. marneffei*. (**a**) Domain architecture of Gst proteins from *T. marneffei* was displayed. The amino acid sequences and putative protein domains were retrieved from Uniprot. Protein domain architectures were generated using IBS web-based tool. (**b**) Putative subcellular localization of 20 TmGsts is depicted. The WoLF PSORT program was used to predict TmGst subcellular localization. mito = mitochondria, cyto = cytoplasm, nucl = nucleus, cysk = cytoskeleton, extr = extracellular space, pero = peroxisome.

Gsts comprise a complex and widespread enzyme family, and therefore we utilized the KEGG pathway map and BLAST program to search for Gst encoding genes. A total of 20 non-redundant gene loci were predicted to encode for putative full-length Gst proteins in *T. marneffei*, which were designated as TmGst1-TmGst20. Sequence characteristics are listed in Table 2 and the domain architectures are presented in Fig. 3a. The sequence lengths of the TmGsts ranged from 164 (TmGst4) to 406 (TmGst13) amino acids. Most of the TmGsts were predicted by the WolF PSORT to localize in cytoplasm (9 out of 20 proteins), followed by mitochondria, and nucleus (Fig. 3b, Table 2, and for full details see Table S2). Our domain analysis using Uniprot, SMART, and conserved domain database (CDD) tools, revealed the presence of two conserved domains, the N-terminal domain (GSH binding site), and the C-terminal domain (substrate binding site) (Fig. 3a). The TmGst4, the TmGst18, and the TmGst20 lacked the Gst C-terminal domain, and the TmGst5 lacked the Gst N-terminal domain. Also, multiple other specific domains were discovered in many TmGst proteins. The TmGst12 was the mitochondrial metaxin-like protein, and therefore uniquely contained the SAM35 (PF10806), the GST N-terminal (PF17172), and the metaxin GST C-terminal (PF17171) domains. This specific GST N-terminal (PF17172) domain was also presented in TmGst20. We found distinct Efb1-gamma domain (PS50040, PF00647) in TmGst13. Besides, the MAPEG (Membrane Associated Proteins in Eicosanoid and Glutathione metabolism) domain (PF01124) was present only in the TmGst16, indicating that the TmGst16 was a putative membrane bound microsomal Gst protein. This was consistent with the subcellular localization prediction of TmGst16 in extracellular space. DSBA-like thioredoxin domain (PF01323), commonly found in the Gst kappa family, was uniquely presented in the TmGst17. The Gst kappa protein family usually presents in mitochondria and peroxisomes, which was consistent with the subcellular localization prediction of TmGst17 in peroxisomes (Fig. 3b and Table 2).

**Figure 4:**
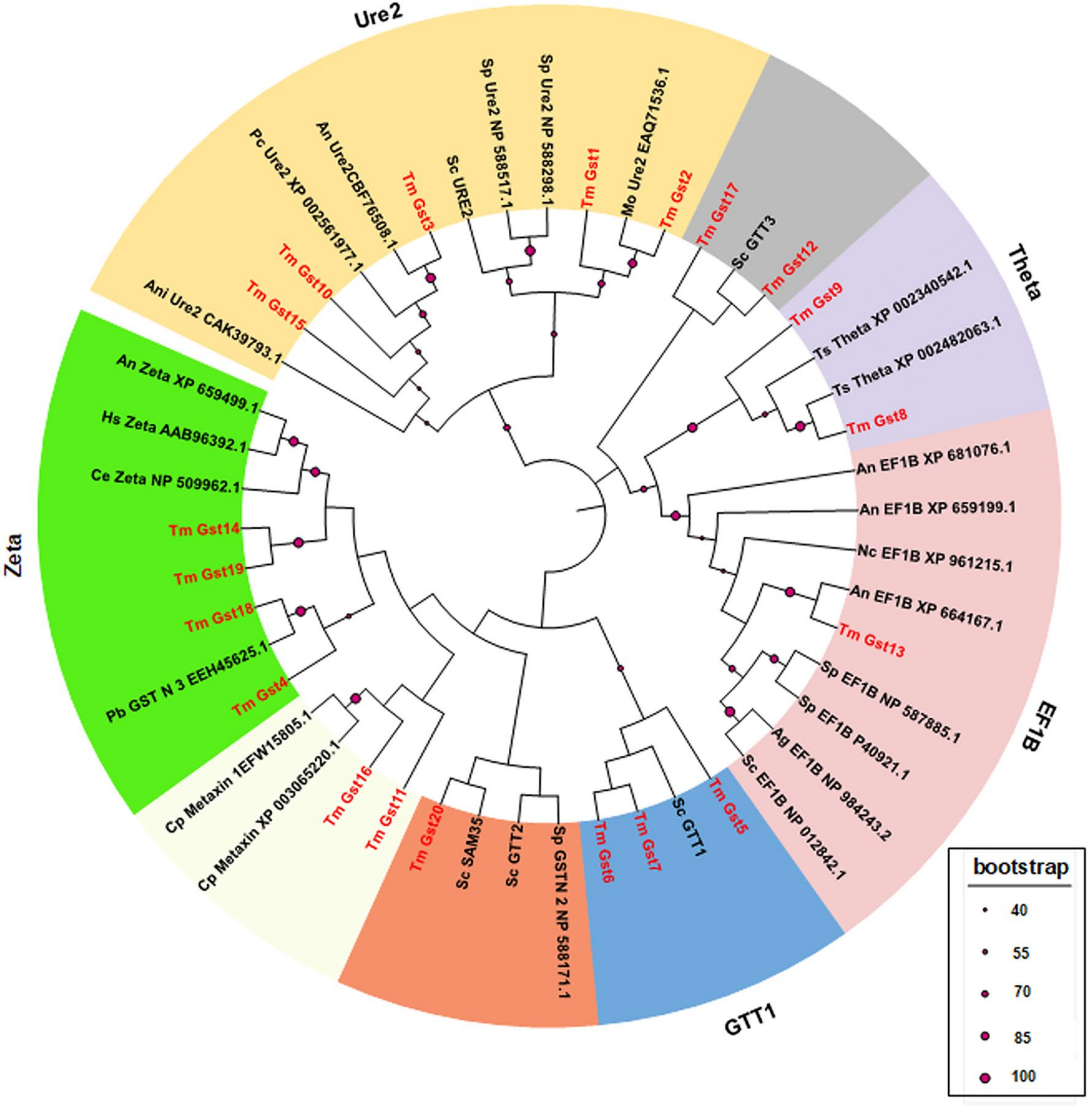
Phylogenetic relationships among the 20 TmGsts. Protein sequences were obtained from BLAST analysis, including 48 proteins from fungi, human and nematodes. The amino acid sequences were aligned using Clustal Omega, and a maximum likelihood tree was constructed using the MEGA 11 software with 500 bootstraps. The red letters represent the Gsts from *T. marneffei*. Magenta circle at nodes of the phylogenetic tree display bootstrap values, and only the values higher than 40 are shown. The accession numbers of all proteins are listed in supplemental data S2.

Gst proteins can be classified based on a variety of criteria, including subcellular localization, amino acid sequences, immunological, kinetics, and structural properties. According to amino acid sequence identities, at least nine classes of cytosolic Gsts have been reported to date in fungi, including GTT1, GTT2, Ure2, MAK16, EFB1, GSTFuA, glutathionyl hydroquinone reductase (GHR), phi, and omega^28,31–33^. To allow for the classification and investigation of the evolutionary relationship among TmGst family members, a total number of 48 full length Gst amino acid sequences from fungi, nematodes and humans were aligned to produce a maximum likelihood phylogenetic tree. Here, based on the constructed phylogenetic tree, multiple clusters can be distinguished (Fig. 4). The tree clearly shows that the largest class of TmGsts is the Ure2-like class (5 members). The Ure2-like class is composed of TmGst1, TmGst2, TmGst3, TmGst10, and TmGst15. The TmGst4, TmGst14, TmGst18 and TmGst19 form a cluster with other Gst Zeta proteins, and therefore the Zeta class constitutes the second largest clade of TmGsts (4 members). The GTT1 class contains TmGst5, TmGst6, and TmGst7. The TmGst8 and TmGst9 are part of the Gst Theta class. The TmGst13 shows a cluster with other fungal EF1B proteins, consistently with its protein domain structures. Overall, the Ure2 and Zeta are the largest classes of TmGsts, and the classification of TmGst proteins is mostly consistent with their protein domain architecture.

### Glutathione metabolic gene expression analysis in different cell types

*T. marneffei* can transit between mold and yeast forms, and this conversion process is thermally regulated^6^. Meanwhile, temperature up-shift is considered as one of the environmental stressors^24^. Heat shock can result in oxidative stress as heat can trigger intensified respiration, leading to elevated intracellular oxidation levels^34,35^. Components of the glutathione-dependent antioxidative system have been shown to participate in heat shock response, including GSH^34,35^, Gcs1 and Gcs2^34,35^, Glr^36,37^, and Gst^38^. Another important cell type is conidium, which is produced from the mold phase and considered as a developmental stage. Accordingly, we aimed to investigate the involvement of *T. marneffei*’s glutathione system in response to temperature-induced morphological change and conidiation by assessing the transcript levels of glutathione genes. To investigate the expression profiles of target genes, conidia from the *T. marneffei* strain ATCC200051 (clinically isolated strain, human infection, 1996) and ATCC18224 (Bamboo rat isolated strain) were either directly harvested or inoculated into Sabouraud’s dextrose broth (SDB) and grown for 72 h at 25 °C or 37 °C to induce the mold and yeast phases, respectively. RNA was collected from these samples, and quantitative real-time PCR was performed to measure gene expression levels.

**Figure 5:**
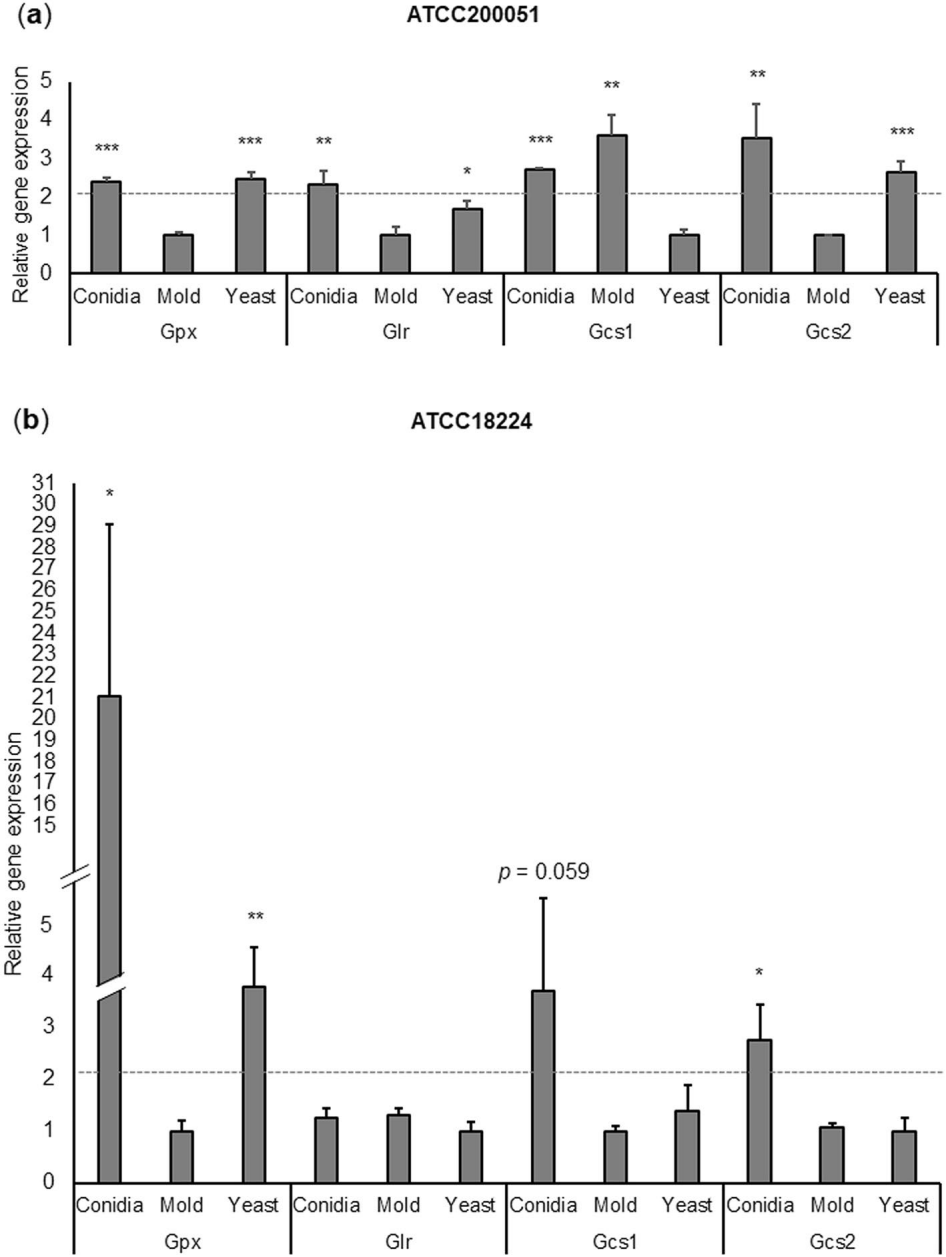
Glutathione metabolic gene expression profile in *T. marneffei* growing in different cell states. Quantitative real-time PCR was performed using RNA extracted from *T. marneffei* growing in conidia, yeast and mold phases. Conidia from the *T. marneffei* ATCC200051 and ATCC18224 strains were either directly harvested or inoculated into Sabouraud dextrose broth and grown for 3 days at 25 °C or 37 °C to induce the mold and yeast phases, respectively (see materials and methods for details). Relative gene expression was calculated by the 2^-∆Ct^ method using actin as a reference gene. Relative fold-change was compared to the phase where transcript was the lowest. Experiments were performed in three biological replicates. Error bars indicate standard deviation. Statistically significant values (*P ≤ 0.05, **P ≤ 0.01, ***P ≤ 0.001) are indicated.

By comparing gene expression patterns from two different strain backgrounds, we were able to identify which glutathione genes were transcriptionally regulated in three cell types: conidia, mold, and yeast. Gene expression analysis showed that the *gpx1* gene was highly expressed in conidia and yeast cells. In ATCC200051 strain, the expression levels of the *gpx1* gene were significantly upregulated by 2.4-fold and 2.5-fold in conidia and yeast cells, respectively, in comparison to the mold phase (Fig. 5a). Consistently, the expression levels of the *gpx1* gene in ATCC18224 strain were significantly upregulated by 21-fold and fourfold in conidia and yeast cells, respectively (Fig. 5b). The *gcs2* gene was significantly upregulated in conidia from two strain backgrounds, being upregulated by 2.5-fold in ATCC200051 strain (Fig. 5a) and 3.5-fold in ATCC18224 strain (Fig. 5b). Our result suggested that, both *T. marneffei* strains transcriptionally regulate the *gpx*1 and *gcs2* genes in conidia and yeast cell types.

Although the *gcs1* and *glr* gene expression levels were not significantly different in ATCC18224 strain, we detected differential expression levels of these genes in the ATCC200051 strain background. As shown in Fig. 5a, the *gcs1* gene was significantly expressed at higher levels, showing a 2.7-fold increase in conidia and a 3.6-fold increase in the mold when compared to the expression levels in the yeast phase. The *glr* gene was significantly upregulated in the conidia and yeast phases, exhibiting a twofold increase in conidia and 1.7-fold increase in the yeast phase when compared to the expression levels in the mold phase. Our results emphasized the difference in transcriptional regulation of glutathione genes between different strain backgrounds. When combining data from two strain backgrounds, we found that most glutathione-related transcripts were highly accumulated in conidia.

To gain insight into the Gst gene expression profile in mold and yeast forms, we initially retrieved gene expression data from DNA microarrays previously performed in mycelial- and yeast-phases of *T. marneffei* cells^9^. In the study by Lin et al., the *T. marneffei* strain B-6323, isolated from a patient with talaromycosis skin lesions, was inoculated into SDB and grown for 48 h at 25 °C or 37 °C to induce the mold and yeast phases, respectively. To facilitate data visualization in our analysis, we converted gene expression data from Lin et al.^9^ into a hierarchical heatmap, using Log2 values of normalized gene expression levels. Sixteen out of twenty TmGsts showed differential gene expression between mold and yeast phases (Fig. S1, Table S3). The TmGst5, TmGst9, TmGst13, and TmGst17 genes were not expressed at different levels (data not shown). Four TmGsts, the TmGst6, TmGst11, TmGst12, and TmGst18, increased their expression levels in the yeast phase, and were clustered together in the heatmap (Fig. S1, highlighted in blue box). The rest of the TmGst encoding genes (twelve TmGsts) were downregulated in the yeast phase (Fig. S1). Among downregulated genes in the yeast phase, the TmGst14 and TmGst3, were the top two Gsts with the greatest decrease in their expression levels, exhibiting a 243-fold and 71-fold downregulation, respectively (Fig. S1, highlighted in the pink box).

**Figure 6:**
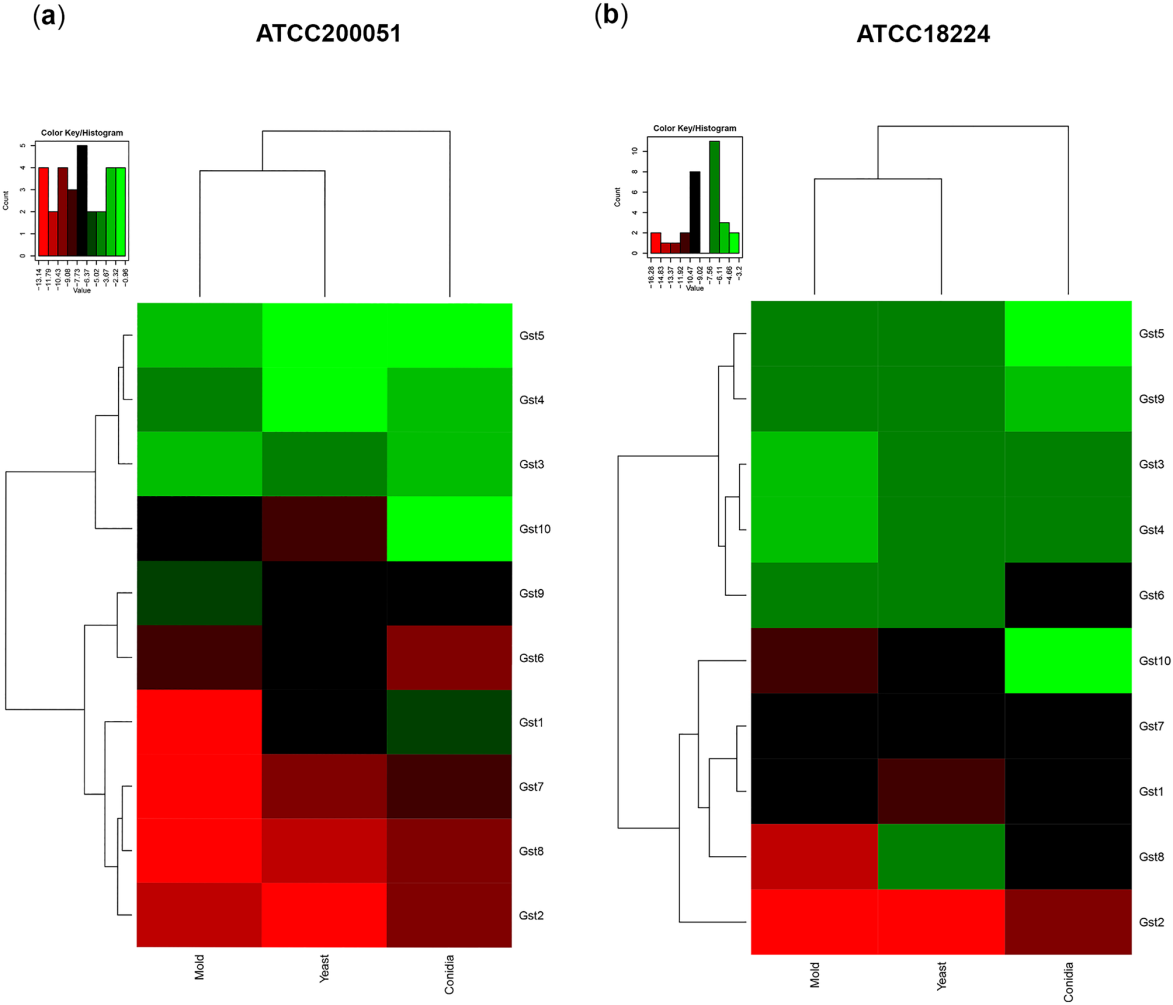
Heatmap depicts the expression patterns of Gst genes from two different strain backgrounds. Gene expression profile was obtained from the *T. marneffei* ATCC18224 (**a**) and ATCC200051 (**b**) strains. The names of the Gst encoding genes are provided on the right side and growth conditions (conidia, mold, and yeast) are provided on the bottom. Dendograms are on the left and on top of the heatmap. Heatmap was generated using the log2 of relative gene expression values (2^−∆Ct^). Strains were grown, RNA was prepared, and gene expression was analyzed as described in the legend of Fig. 5. Experiments were performed in three biological replicates.

To further determine which Gst members exhibited phase specific expression, we experimentally examined the transcripts of the first 10 Gsts, i.e., TmGst1-TmGst10, using RNA samples prepared from conidia and the 72-h mold and yeast cultures of *T. marneffei* ATCC200051 and ATCC18224 strains. The hierarchical heatmap was generated (Fig. 6). As depicted in Fig. 6, conidia exhibited a unique gene expression profile, forming a separate cluster from the mold and yeast phases. The TmGst2 and TmGst10 showed high expression levels in conidia. The TmGst10 gene exhibited conidial specificity by showing a 220-fold upregulation in ATCC200051 strain and a 300-fold upregulation in ATCC18224 strain, respectively (Figs. 6 and S2). The TmGst2 gene had the least abundant transcript yet showed over a tenfold increased accumulation in conidia from both strain backgrounds (Figs. 6 and S3). The TmGst6 gene expression was upregulated in the yeast phase, exhibiting a 12-fold and ninefold increase in ATCC200051 and ATCC18224 strains, respectively, when compared to gene expression levels detected in conidia (Figs. 6 and S4). The TmGst3 gene was expressed at higher levels in the mold phase, showing a fourfold and threefold increase in ATCC200051 and ATCC18224 strains, respectively (Figs. 6 and S5). Considering gene expression data previously performed (Lin et al.^9^) and our current data, the TmGst6 and the TmGst3 genes consistently exhibited similar gene expression profiles across three different tested strain backgrounds. Specifically, the TmGst6 was upregulated in the yeast phase and the TmGst3 was upregulated in the mold phase. Our study also suggested that the TmGst10 transcript was highly accumulated in conidia. These results indicate that each Gst plays different roles in different cell types of *T. marneffei*.

In summary, most glutathione metabolic genes were transcriptionally regulated when *T. marneffei* underwent thermal-induced morphological changes or conidiation (Table 3). Our results underlined that the functions of glutathione-metabolic proteins could be dependent on strain backgrounds and morphological forms. Several glutathione genes consistently exhibited similar expression patterns across tested strain backgrounds, and we postulated that these genes encode the major enzymes, functioning in a cell type-specific manner.

**Table 3:** Summary of glutathione gene expression patterns in *T. marneffei* from two different strain backgrounds.

Enzyme	Gene	Morphology	Relative fold-change#	
			ATCC200051	ATCC18224
Glutamate cysteine ligase	*Gcs1*	Conidia Mold Yeast	2.70 3.60 1.00	3.68 1.00 1.38
Glutathione synthetase	*Gcs2*	Conidia Mold Yeast	3.54 1.00 2.63	2.74 1.05 1.00
Glutathione peroxidase	*Gpx1*	Conidia Mold Yeast	2.41 1.00 2.47	21.06 1.00 3.77
Glutathione reductase	*Glr*	Conidia Mold Yeast	2.32 1.00 1.69	1.26 1.31 1.00
Glutathione S-transferase	*Gst1*	Conidia Mold Yeast	142.89 1.00 70.27	2.68 2.00 1.00
	*Gst2*	Conidia Mold Yeast	12.18 4.54 1.00	16.63 1.00 2.45
	*Gst3*	Conidia Mold Yeast	2.26 3.87 1.00	1.24 3.36 1.00
	*Gst4*	Conidia Mold Yeast	1.48 1.00 3.55	1.00 2.99 1.56
	*Gst5*	Conidia Mold Yeast	1.99 1.00 1.29	11.34 1.00 2.27
	*Gst6*	Conidia Mold Yeast	1.00 2.91 12.35	1.00 6.15 8.84
	*Gst7*	Conidia Mold Yeast	24.59 1.00 10.24	1.72 2.00 1.00
	*Gst8*	Conidia Mold Yeast	8.41 1.00 2.19	17.56 1.00 89.99
	*Gst9*	Conidia Mold Yeast	1.00 2.16 1.17	2.05 1.00 1.32
	*Gst10*	Conidia Mold Yeast	220.12 4.04 1.00	299.75 1.00 3.68

^**#**^Fold change data = cell type/cell type with lowest gene expression ratios (For example, yeast/conidia where target gene was expressed at the lowest levels in conidia).

### The Gst gene expression profile in response to xenobiotics

**Figure 7:**
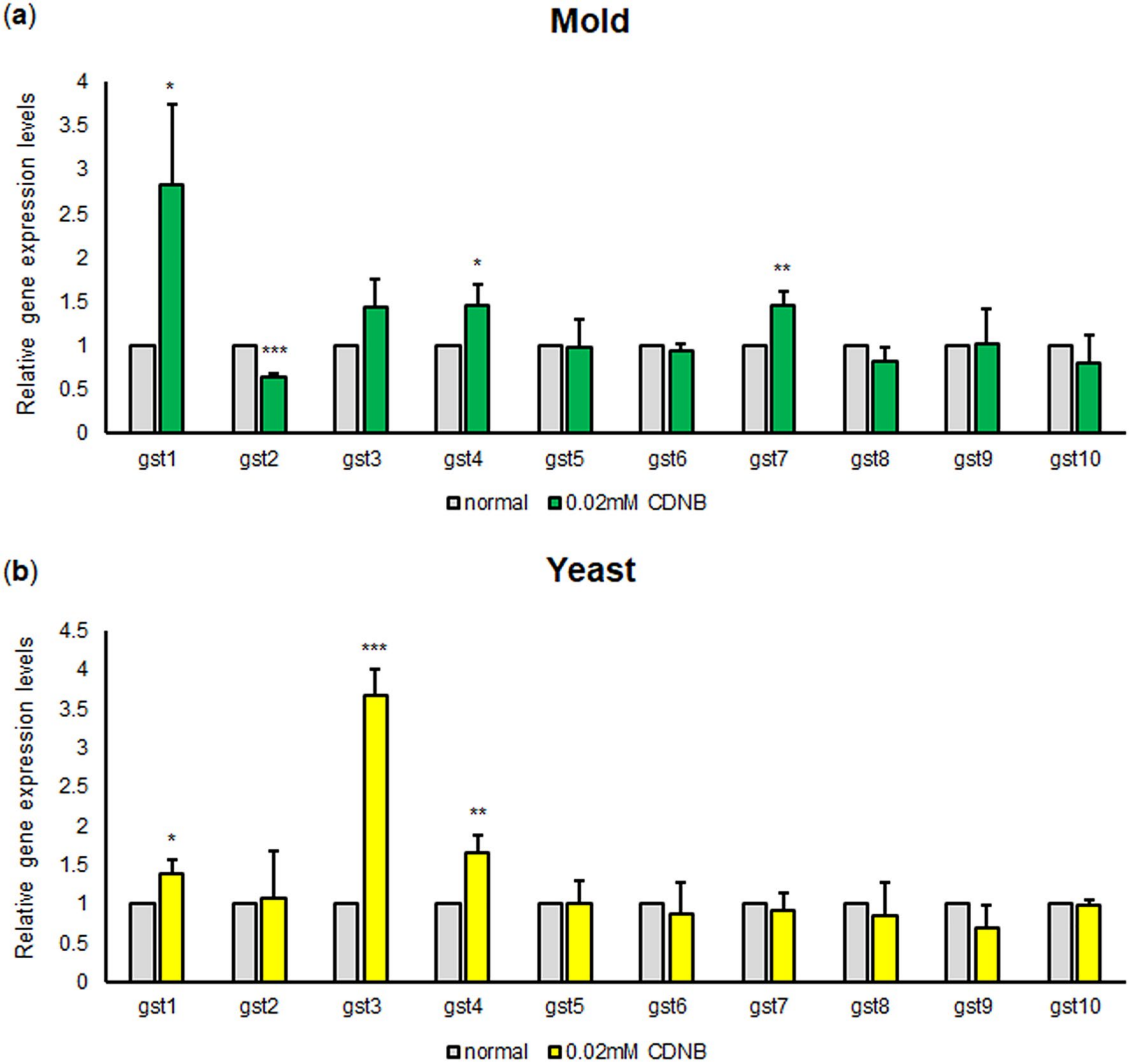
Glutathione-S transferase gene expression profile in *T. marneffei* growing under xenobiotics (CDNB) treatment. Conidia from the *T. marneffei* ATCC200051 strain were inoculated into Sabouraud dextrose broth and grown for 3 days at 25 °C or 37 °C to induce either the mold (**a**) or yeast phase (**b**), respectively. *T. marneffei* cultures were either directly harvested (no treatment control/normal) or treated with CDNB (0.02 mM) and harvested at 60 min after treatment. RNA samples were extracted and subjected to cDNA synthesis and quantitative real-time PCR. The experiment was performed in three biological replicates. Error bars indicate standard deviation. Selected Gsts are the TmGst1-10. Statistically significant values (*P ≤ 0.05, **P ≤ 0.01, ***P ≤ 0.001, ns = not significant) are indicated.

The GST proteins play critical roles in the detoxification of xenobiotics, which are defined as foreign compounds that are not naturally produced within the organism. Recently, antifungal drugs are considered as important xenobiotics for *T. marneffei* and drug resistance is a major medical problem worldwide. Based on this public health concern, we reasoned that Gsts might participate in the detoxification of foreign substances, which could ultimately contribute to the antifungal drug resistance mechanism. To investigate if the *T. marneffei* GSTs were involved in the xenobiotic response, the synthetic substrate 1-chloro-2, 4-dinitrobenzene (CDNB) was selected as the xenobiotic model because it is the substrate for most GST enzymes. As an initial step, we analyzed if the Gst genes could be transcriptionally regulated in response to xenobiotics. We treated the mold and yeast cultures of *T. marneffei* strain ATCC200051 with CDNB (0.02 mM) for 1 h and measured the transcript levels of TmGst1 to TmGst10. In the mold phase, the TmGst1 showed the highest fold increase, being upregulated by almost threefold when compared to untreated samples (Fig. 7a). In the yeast phase, the TmGst3 showed the highest fold upregulation, exhibiting a 3.5-fold elevation in exposure to CDNB (Fig. 7b). Our data indicated that the TmGst1 and TmGst3 were transcriptionally responsive to CDNB in a phase-specific manner.

### Glutathione metabolic gene expression analysis under oxidative stress

**Figure 8:**
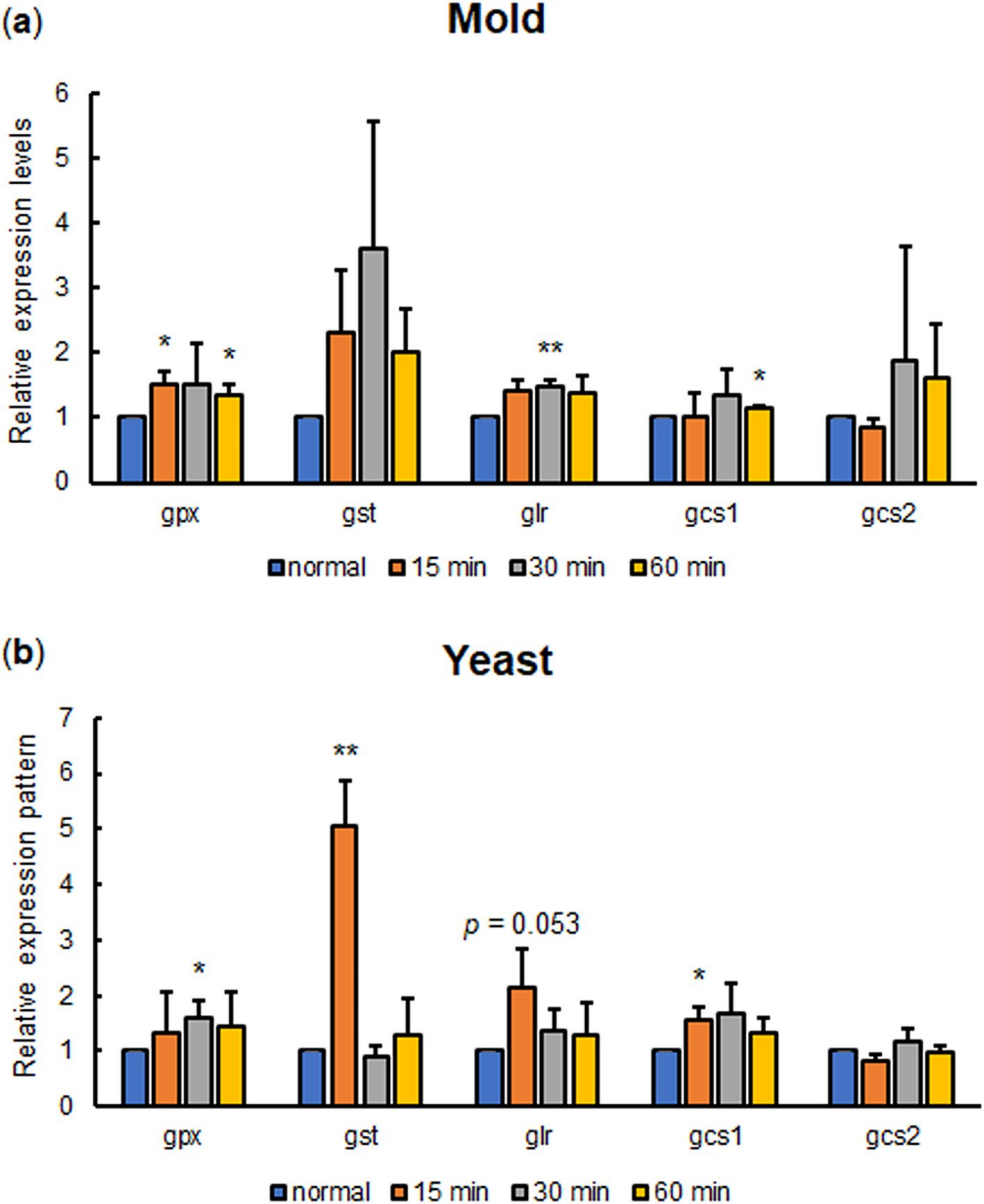
Glutathione metabolic gene expression profile in *T. marneffei* growing under oxidative stress conditions. Conidia from the *T. marneffei* ATCC200051 strains were inoculated into Sabouraud dextrose broth and grown for 3 days at 25 °C or 37 °C to induce either the mold (**a**) or yeast (**b**) phase, respectively. *T. marneffei* cultures were either directly harvested (no treatment control/normal) or were treated with H_2_O_2_ (2 mM), and harvested at 15, 30 and 60 min after treatment. RNA samples were extracted and subjected for cDNA synthesis and quantitative real-time PCR. Experiments were performed in three biological replicates. Error bars indicate standard deviation. Selected Gst is the TmGst3. Statistically significant values (*P ≤ 0.05, **P ≤ 0.01, ***P ≤ 0.001, ns = not significant) are indicated.

During host colonization and invasion, *T. marneffei* is exposed to external oxidative stress, produced by the host macrophages. To determine if the *T. marneffei* glutathione system participated in cellular response to an external oxidative stressor, the 72-h mold and yeast cultures of *T. marneffei* ATCC200051 strain were exposed to 2 mM H_2_O_2_. After 15, 30, and 60 min of H_2_O_2_ treatment, cultures were collected, and gene expression levels were analyzed. For Gst genes, we selected to assess the transcript levels of TmGst3 among the other 20 Gst proteins because it consistently exhibited phase-specific expression in our report and in the study by Lin et al. Strikingly, the TmGst3 in yeast phase cultures showed the highest gene upregulation at 15 min after H_2_O_2_ exposure, having a fivefold increase compared to the no treatment control. (Fig. 8a). However, at 30 min and 60 min after H_2_O_2_ exposure, the TmGst3 expression levels were no longer elevated. In addition, the *gpx1* and *gcs1* transcripts were significantly increased in response to H_2_O_2_ treatment. This result revealed that the genes in glutathione biosynthesis, recycling and detoxification responded quickly to the stress conditions within the first 15–60 min (Fig. 8). Overall, our results demonstrated that *T. marneffei* generally induced the expression of glutathione genes when the cells encountered oxidative stress.

## Discussion

Glutathione and glutathione metabolic proteins play a key role in the response to a myriad of stress situations in fungi^27^. However, the contributions of this glutathione system are mostly uncharacterized in thermal dimorphic fungi^39^. As an intracellular pathogen, *T. marneffei* has to alleviate the stress generated in the phagosomal environment. Previous studies reported the role of enzymatic antioxidants in *T. marneffei*, including superoxide dismutase and catalase-peroxidase; however, only a few studies have characterized the glutathione gene expression profiles^19,40^. Indeed, glutathione gene disruption studies have never been performed in this fungal species. Using the NCBI and Uniprot databases, we identified *T. marneffei* genes encoding the major enzymes of glutathione biosynthesis, glutathione recycling, and glutathione-related detoxification. To understand the function of glutathione metabolism in different cell types, we measured the expression levels of glutathione metabolic genes when *T. marneffei* exists in yeast, mold, and the infectious asexual conidia. Several gene expression patterns were discovered in our analyses. First, the transcript levels of glutathione biosynthetic gene TmGcs2 were detected in conidia of *T. marneffei* from two different strain backgrounds and in the thermal-induced yeast form of *T. marneffei* ATCC200051 strain. Likewise, the *GSH2* gene (the Gcs2 homolog) from the dimorphic human fungal pathogen *H. capsulatum* was highly expressed in the yeast form compared to the mold form, consistent with our result^41^. In *S. cerevisiae*, the expression of both *GSH1* and *GSH2* genes was induced under heat shock treatment (41 °C)^34,35^. As mentioned previously, elevated temperature from 25 to 37 °C is a critical inducer that triggers yeast morphology in *T. marneffei* and *H. capsulatum*. Together, these results suggest that the glutathione biosynthesis pathway is responsive to heat-induced stress that might occur during morphological conversion in dimorphic fungi.

Second, the glutathione peroxidase gene was highly accumulated in conidia and upregulated in the yeast form of *T. marneffei* from both ATCC200051 and ATCC18224 strains but was not inducible under H_2_O_2_ exposure. While other fungal species have multiple gene copies encoding glutathione peroxidase, there is only one glutathione peroxidase homolog in *T. marneffei*^39^. For example, in the yeast *S. cerevisiae*, there are three glutathione peroxidase homologs, *GPX1*, *GPX2,* and *HYR1* (also known as *GPX3* and *ORP1*). In *C. albicans*, there are four homologs of glutathione peroxidase, *GPX3* (homolog of *S. cerevisiae GPX1*), *GPX31*, *GPX32*, and *GPX33* (*GPX31-33* are homolog of *ScHYR1*)^42,43^. In *S. cerevisiae*, the expression levels of *HYR1* gene were constitutively high and not inducible by any tested stressors^44^. Yet, the Sc*GPX1* gene was induced by glucose starvation while the Sc*GPX2* gene was induced by oxidative stress^44^. In *A. fumigatus*, the *hyr1* gene was moderately induced upon H_2_O_2_ treatment (fold-change < 2, *p*-value < 0.05;^45^. In *C. albicans*, only the *GPX31* gene was highly induced upon exposure to oxidative stress^43^. These studies together demonstrate differences in transcriptional regulation of each glutathione peroxidase, which could be specific to certain stressors and could be varied among fungal species.

Gsts are a family of multifunctional enzymes that play a major role in stress response and detoxifying a wide range of endogenous and exogenous compounds. In the present study, a comprehensive genome-wide search identified a total of 20 TmGst gene members. This massive expansion of Gsts in *T. marneffei* is in agreement with the diversification of Gst proteins found in fungi and other organisms^33^. Indeed, the term “GSTome” has been coiled to reflect the entire collection of all Gsts and their roles in an organism^46^. In fungi, the Gst isoform number is ranged from 4 gene copies in the basidiomycete yeast *Sporobolomyces roseus* to 44 gene copies in brown rot fungi *Postia placenta* and *Coniophora puteana*^33^. Notably, there is no correlation between the trophic mode of the fungi (e.g., saprophytic, symbiotic, and pathogenic modes) and their Gst numbers^33^.

Based on the evolutionary analysis, we were able to classify most of the TmGsts into the known Gst class with high bootstrap values. The Ure2 class is composed of TmGst1, TmGst2, TmGst3, TmGst10, and TmGst15. The GTT1 class is composed of TmGst5, TmGst6, and TmGst7. The Theta class is composed of TmGst8 and TmGst9. The EF1B class is composed of TmGst13. The Zeta class is composed of TmGst4, TmGst14, TmGst18, and TmGst19. However, it is generally known that the classification of fungal GSTome was difficult due to (i) the limitation of standard classification criteria and (ii) the increase in fungal Gst numbers and functions. In fact, some of the TmGsts do not fit easily into the previously characterized classes of fungi^28,47^. In our study, for example, the MAPEG TmGst16 was placed in the Metaxin1/SAM35 class with lower bootstrap values even though they contain different functional domains.

In multicellular organisms, Gsts display functional specificity to organs and tissues, underlying their necessity in growth and development^48–51^. In *T. marneffei*, the TmGst gene expression levels were altered in a cell state-specific manner^9,10^ (This study). The TmGst6 gene was highly upregulated in temperature-induced yeast form^9^ (This study). This TmGst6 gene induction in the yeast form is consistent in three different strain backgrounds, strengthening the prominent function of TmGst6 in pathogenic yeast cells. Multiple gene expression studies demonstrated that the TmGst3^9^ (This study) and TmGst14^9,10^ genes were downregulated in the yeast form (i.e., highly expressed in mold form), suggesting that TmGst3 and TmGst14 proteins are important in mold phase. Moreover, the TmGst10 gene showed the highest accumulation in conidia of *T. marneffei* strain ATCC20051 and ATCC18224 (This study). The rest of the tested TmGst genes exhibited strain background-specific expression profiles. Taken together, our identification of 20 TmGsts and characterization of their gene expression profiles reinforced the common roles of Gst in growth and development at both cellular and tissue/organ levels^48–51^.

The expression levels of Gst genes usually change in response to specific substrates or stressors. In exposure to hydrogen peroxide, we found that TmGst3 was highly upregulated in the yeast phase in comparison to the mold phase or to other glutathione metabolic genes. This result is consistent with the induction of glutathione S-transferase gene homologs found in other fungal species. In *S. cerevisiae*, the *GTT1* and *GTT2* genes were induced during the diauxic shift and stationary phase where the yeast cells accumulated more ROS levels, and hence experienced more oxidative stress^38^. In *S. pombe*, the *gst1*^+^, *gst2*^+^, and *gst3*^+^ genes were upregulated during the stationary phase and in response to hydrogen peroxide^52^. The *gstA*, *gstB,* and *gstC* genes from *A. fumigatus* and the *gstA* gene from *A. nidulans* were all induced in response to hydrogen peroxide treatment^53,54^. Besides the role in oxidative stress response, many fungal Gst encoding genes are also upregulated in response to heavy metals, xenobiotics, or other stress conditions. For example, the *GST2* gene expression from *C. albicans* is induced under nitrogen starvation^55^ while the Gst genes from *Aspergilli* were induced in response to xenobiotic CDNB^53,54^. Our result was consistent with these studies as we could detect the upregulated levels of the TmGst1 gene in the mold phase and TmGst3 in the yeast phase after CDNB treatment. Various mechanisms are involved in antifungal drug resistance. Nonetheless, insights into the detoxification effect of Gsts on antifungal drugs are limited. In *Fusarium graminearum*, the mutant that was resistant to benzimidazole showed higher Gst activity than the non-resistant strain^56^. This data strongly suggests that Gst might participate in antifungal drug resistance in *F. graminearum*. In *T. marneffei*, TmGst1 and TmGst3 were transcriptionally upregulated in response to CDNB, suggesting that specific types of TmGst proteins can bind and detoxify the CNDB. Presumably, other types of TmGst could play a role in detoxifying other xenobiotics, including antifungal drugs. Future experiments will be needed to determine which type of TmGsts are implicated in antifungal drug detoxification and drug resistance.

Our data suggest that glutathione metabolic genes are transcriptionally regulated to maintain their specialized or ubiquitous functions. In *S. cerevisiae*, Yap1 is a transcription factor that directly upregulates the expression of *GSH1*, *GSH2*, *GLR*, and *GPX2* genes^35,37,57,58^. ScHyr1 acts as a hydroperoxide sensor, forming a disulfide bond with Yap1, and thereby masking a Yap1 nuclear export signal^59,60^. This interaction leads to the retention of Yap1 in the nucleus to upregulate oxidative responsive genes^61,62^. In *S. pombe*, the transcription factor Pap1 (ScYap1 homolog) and Atf1 control the transcriptional regulation of glutathione metabolic genes under stress conditions^63,64^. The expression of *gst1*^+^ and *gst2*^+^ is Pap1-dependent, whereas the induction of *gst*3^+^ is Atf1-dependent^52^. In *T. marneffei*, the upregulation of TmGcs1 and TmGlr genes in response to oxidative stressors H_2_O_2_, menadione and NaNO_2_ are dependent on yapA (Yap1 homolog) in all growth forms (conidia, mold, and yeast)^40^. Besides TmGcs1 and TmGlr genes, other glutathione metabolic genes were not tested in the study by Dankai et al. Additionally, while the *atfA* (SpAtf1 homolog) deletion mutant in *T. marneffei* shows sensitivity to various oxidative stressors, whether glutathione metabolic gene expression is dependent on aftA was not tested^65^. With available data, we hypothesized that the yapA or/and atfA are likely the key transcription factors that induce the expression of glutathione metabolic genes reported in our current studies. More investigation is needed to fully decipher mechanisms that regulate the transcription of glutathione homeostasis genes in *T. marneffei*.

## Conclusion

In conclusion, we characterized the glutathione metabolic system in the thermal dimorphic fungus *T. marneffei*. Using the homologous sequence search, we identified glutathione biosynthetic enzymes (Gcs1 and Gcs2), glutathione peroxidase (Gpx1), glutathione reductase (Glr1), and a family of glutathione S-transferases (Gsts). There are 20 TmGsts (TmGst1-20), and phylogenetic analysis revealed the evolutionary conservation of TmGsts with other classes of known fungal Gst proteins. Based on the evolutionary relationship, the TmGsts were divided into at least 4 classes, including the Ure2, the GTT1, EF1B, and Zeta. Comprehensive gene expression analysis was performed in three different cell types, including conidia, mold, and temperature-induced yeast cells. Most glutathione metabolic genes exhibited differential gene expression patterns when grown in different cell phases. As the ability of *T. marneffei* to change morphology and to survive the macrophage killing process is a key virulence factor, regulation of the glutathione metabolic system could potentially facilitate *T. marneffei* survival and dimorphic switching during host invasion and infections. Thus, this study will aid in the understanding of pathobiology of *T. marneffei*, and help researchers identify phase-specific genes/biomarkers.

## Materials and methods

### Fungal strains and gene expression analysis

*T. marneffei* strain ATCC20051 (CBS119456) and FRR2161 (ATCC18224) were cultured on Potato Dextrose Agar (PDA) at 25 °C for 10–14 days to generate conidia. The conidia were harvested and 1 × 10^8^ conidia/ml were inoculated in a 50-ml Sabouraud’s dextrose broth (SDB). Cultures were incubated either at 25 °C (mold phase) or 37 °C (yeast phase) with continuous shaking at 200 rpm. After 72 h, cultures were collected by centrifugation at 4 °C, 7000 rpm for 30 min. For oxidative stress, cultures in mold and yeast phases were grown for 72 h, and 2 mM H_2_O_2_ was added for 15 min, 30 min, and 1 h with continuous shaking at 200 rpm. For xenobiotic treatment, cultures in mold and yeast phases were grown for 72 h, and 0.02 mM 1-chloro-2,4-dinitrobenzene (CDNB) (Sigma-Aldrich, City, Country) was added for 1 h with continuous shaking at 200 rpm. After indicated incubation time, cells were harvested by centrifugation and subjected to gene expression analysis.

Total RNA was isolated by using TRIzol® reagent, treated with DNase I, and converted to cDNA as previously described^66^. Quantitative real-time PCR was performed using the SYBR Green qPCR mix (Thunderbird SYBR Green Chemistry, TOYOBO. Osaka, Japan). An actin gene was included as a reference gene (internal control). All primers used in this study are listed in Table S1. Calculation of a relative expression was performed using the 2^−(∆Ct)^ where ∆C_t_ = C_t_ actin − C_t_ target). The Student’s *t*-test and statistical significance were calculated by using Excel. Error bars indicate standard deviation, calculated by using Excel.

### Heatmap generation

Heatmap indicating transcript abundance and expression pattern was generated using the HEATMAP hierarchical clustering web tool (https://www.hiv.lanl.gov/content/sequence/HEATMAP/heatmap.html). The log2 fold-change values of relative expression levels (the 2^−(∆Ct)^ value) were used to construct the heatmap.

### Bioinformatic analysis

DNA sequences of glutathione genes (*gcs1*, *gcs2*, *gpx1*, *glr1*, and *gst*) from the yeast models *Saccharomyces cerevisiae* were submitted to the basic local alignment search tool for nucleotides (BLASTn) to search for sequence similarity against *T. marneffei* strain ATCC18224. Amino acid sequences of identified glutathione genes from *T. marneffei* and other fungal homologs were obtained from the BLAST search tool for protein (BLASTp). Functional domains and signature patterns were predicted using the Prosite (https://prosite.expasy.org/scanprosite/), SMART (http://smart.embl-heidelberg.de/), KEGG (https://www.kegg.jp/kegg/kegg1.html), and Uniprot (https://www.uniprot.org/) tools. Visualization of the Gst functional domains was performed using the IBS 2.0 tool^67^ (https://ibs.renlab.org). The compute pI/Mw tool was used to predict molecular weight (Mw) and isoelectric points (PI) of Gst proteins (https://web.expasy.org/compute_pi/). The presence of secretory signal sequences and protein subcellular localization were predicted using the SignalP and WoLF PSORT programs^68,69^.

The Clustal omega program was employed to perform protein sequence alignment (https://www.ebi.ac.uk/Tools/msa/clustalo/). Phylogenetic trees were constructed in MEGA 11, by the Neighbor-joining method using 1000 bootstraps as the phylogeny test for Gpx proteins, and by the Maximum Likelihood method using 500 bootstraps as the phylogeny test for Gst proteins. The tree was further decorated using the web-based tool iTOL: Interactiv Tree of Life^70^ (https://itol.embl.de/).

### Supplementary Information

Supplementary Figures.

Supplementary Table S1.

Supplementary Table S2.

Supplementary Table S3.

Supplemental Information 5.

Supplemental Information 6.

## Data Availability

Gene expression datasets generated and analyzed during the current study are deposited and available at Figshare (https://doi.org/10.6084/m9.figshare.23265335). Primer sequences are provided in Table S1. Data used to generate a heatmap in Fig. S1 are freely accessible at https://doi.org/10.3109/13693786.2012.678398, and only specific data of Gst gene expression are summarized in Table S3.
