# Supplementary material for: Identification of glutathione metabolic genes from a dimorphic fungus Talaromyces marneffei and their gene expression patterns under different environmental conditions
Source: Sci Rep. 2023 Aug 24;13:13888. doi: 10.1038/s41598-023-40932-w (PMC10449922; doi:10.1038/s41598-023-40932-w)
Supplement: Supplementary file 1 — Supplementary Figures. [file 41598_2023_40932_MOESM1_ESM.pdf]

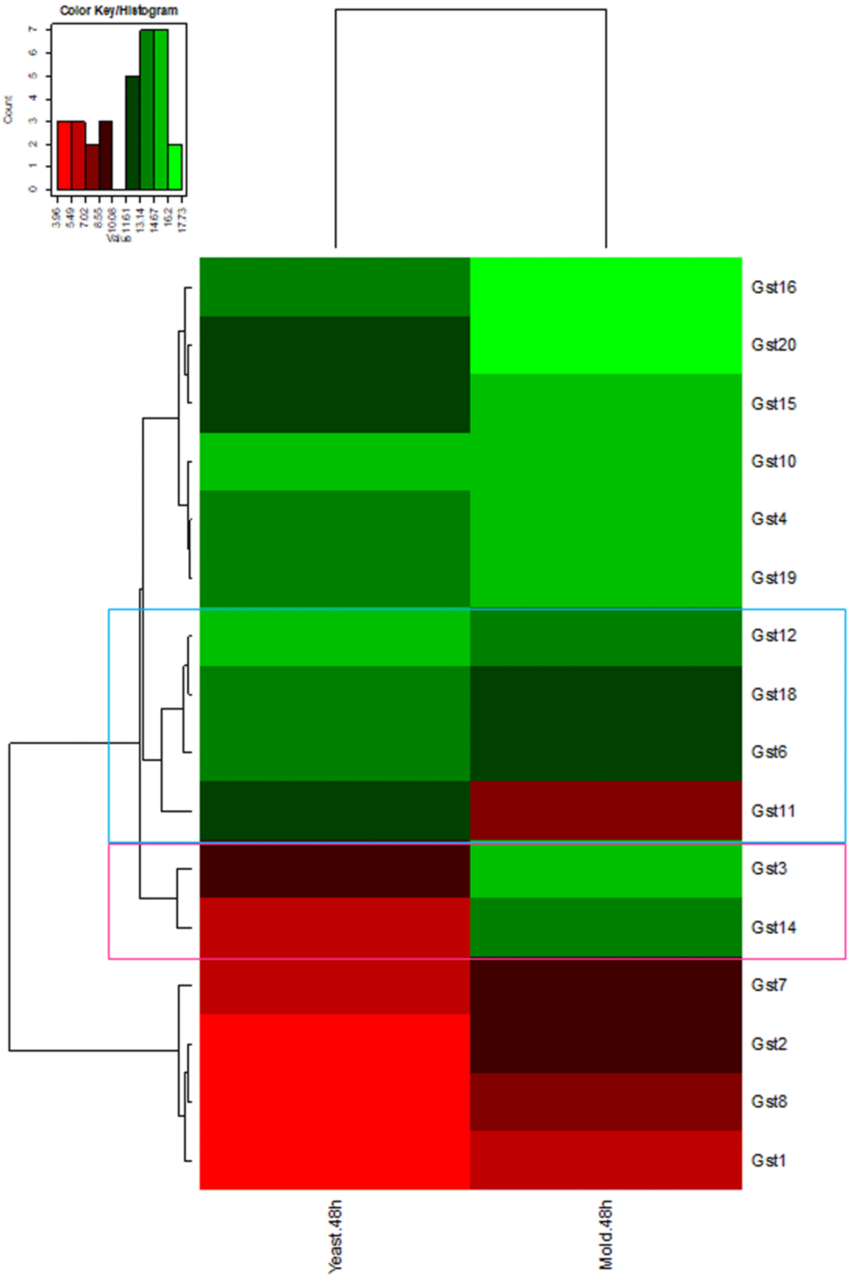

Figure S1. Heatmap depicts the expression patterns of Gst genes from the B-6323 strain background. The Log2 normalized values were regenerated from gene expression levels retrieved from DNA microarray data by Lin et al. 2012. The names of the Gst encoding genes are provided on the right side and growth conditions (conidia, mold, and yeast) are provided on the bottom. Dendograms are on the left and on top of the heatmap.

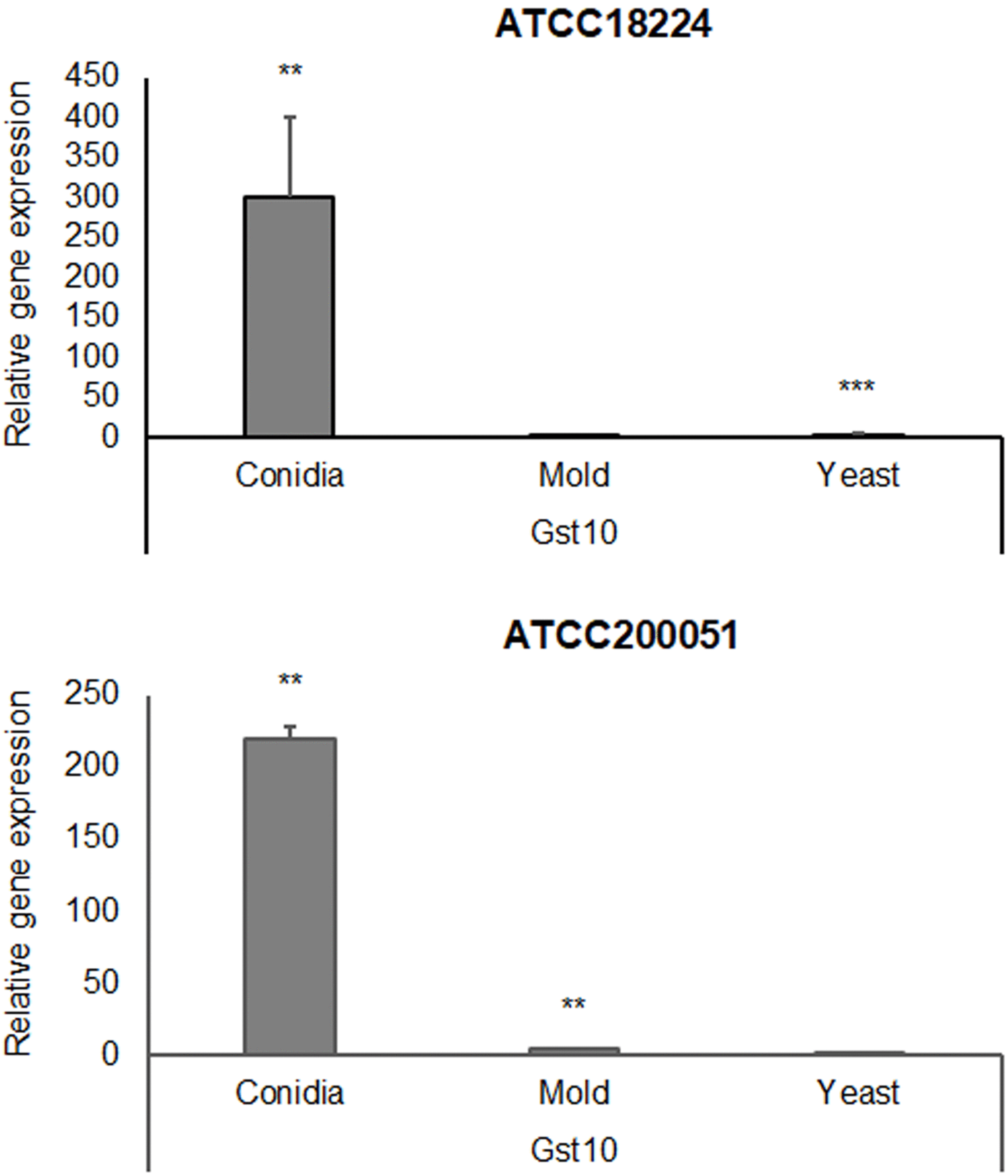

Figure S2. Glutathione S-transferase gene expression profile in *T. marneffei* growing in different cell states. Strains were grown, RNA was prepared, and gene expression was analyzed as described in the legend of Figure 5. Experiments were performed in three biological replicates. Error bars indicate standard deviation. Statistically significant values (* $P \leq 0.05$, ** $P \leq 0.01$, *** $P \leq 0.001$) are indicated.

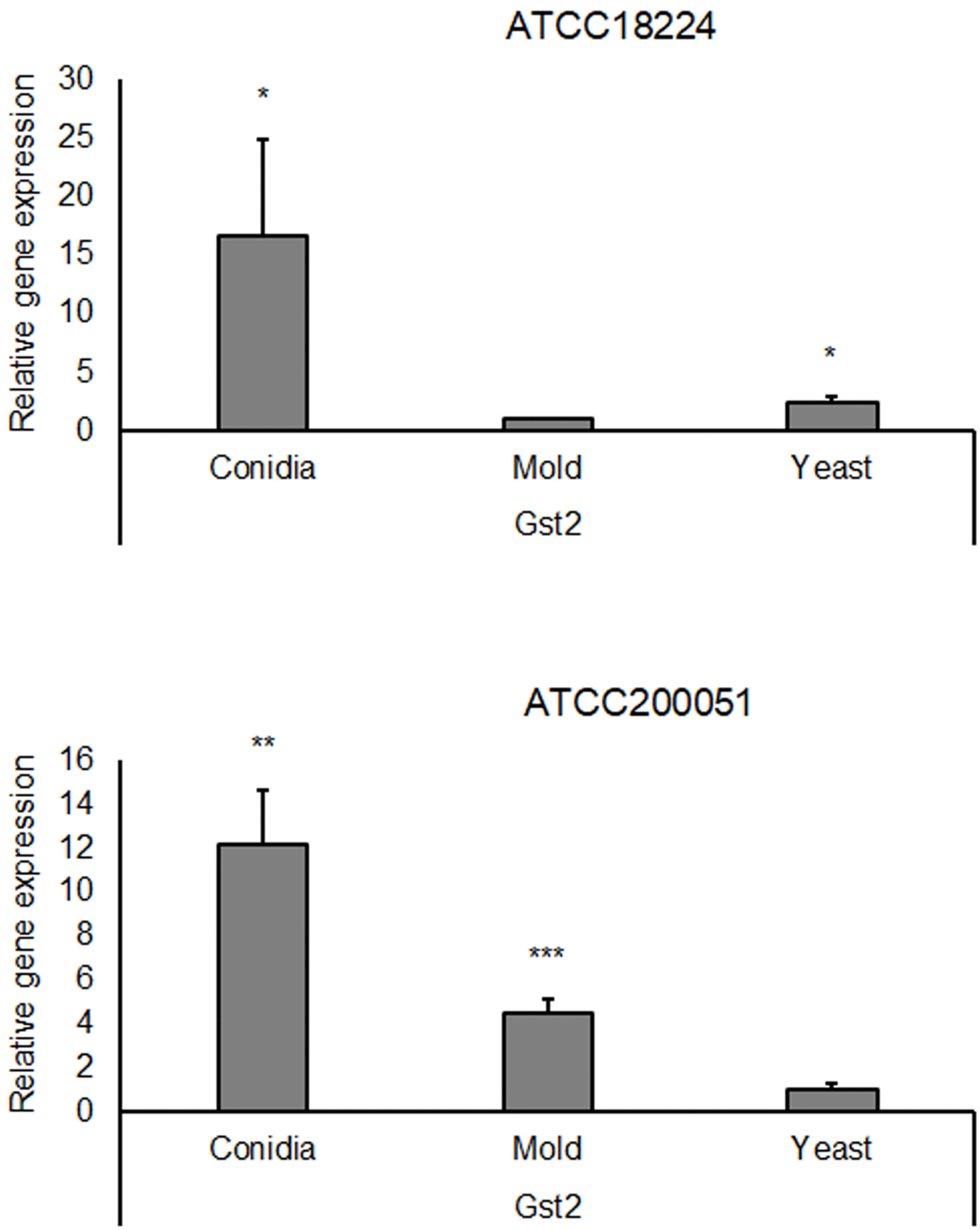

Figure S3. Glutathione S-transferase gene expression profile in *T. marneffei* growing in different cell states. Strains were grown, RNA was prepared, and gene expression was analyzed as described in the legend of Figure 5. Experiments were performed in three biological replicates. Error bars indicate standard deviation. Statistically significant values (* $P \leq 0.05$, ** $P \leq 0.01$, *** $P \leq 0.001$) are indicated.

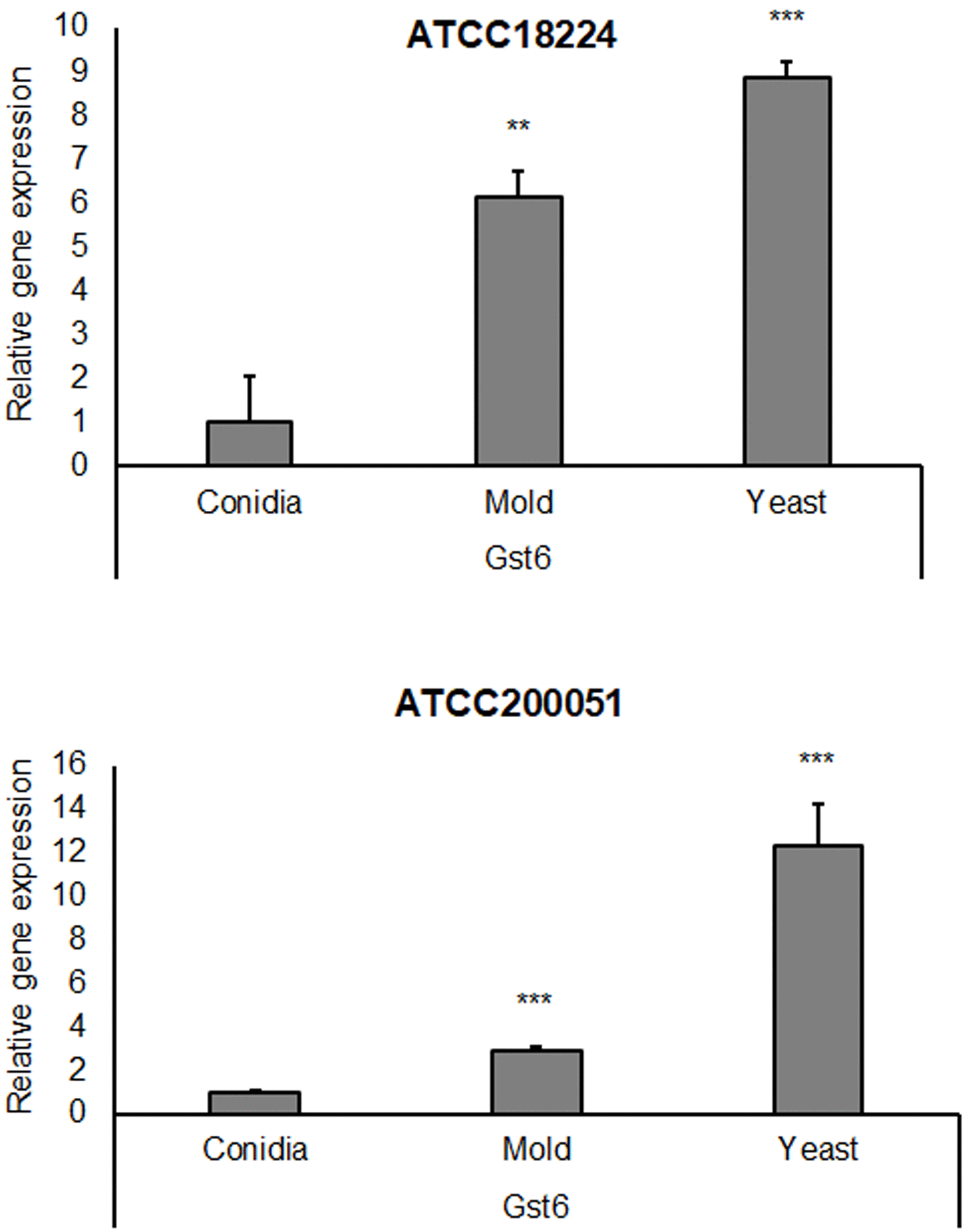

Figure S4. Glutathione S-transferase gene expression profile in *T. marneffei* growing in different cell states. Strains were grown, RNA was prepared, and gene expression was analyzed as described in the legend of Figure 5. Experiments were performed in three biological replicates. Error bars indicate standard deviation. Statistically significant values (* $P \leq 0.05$, ** $P \leq 0.01$, *** $P \leq 0.001$) are indicated.

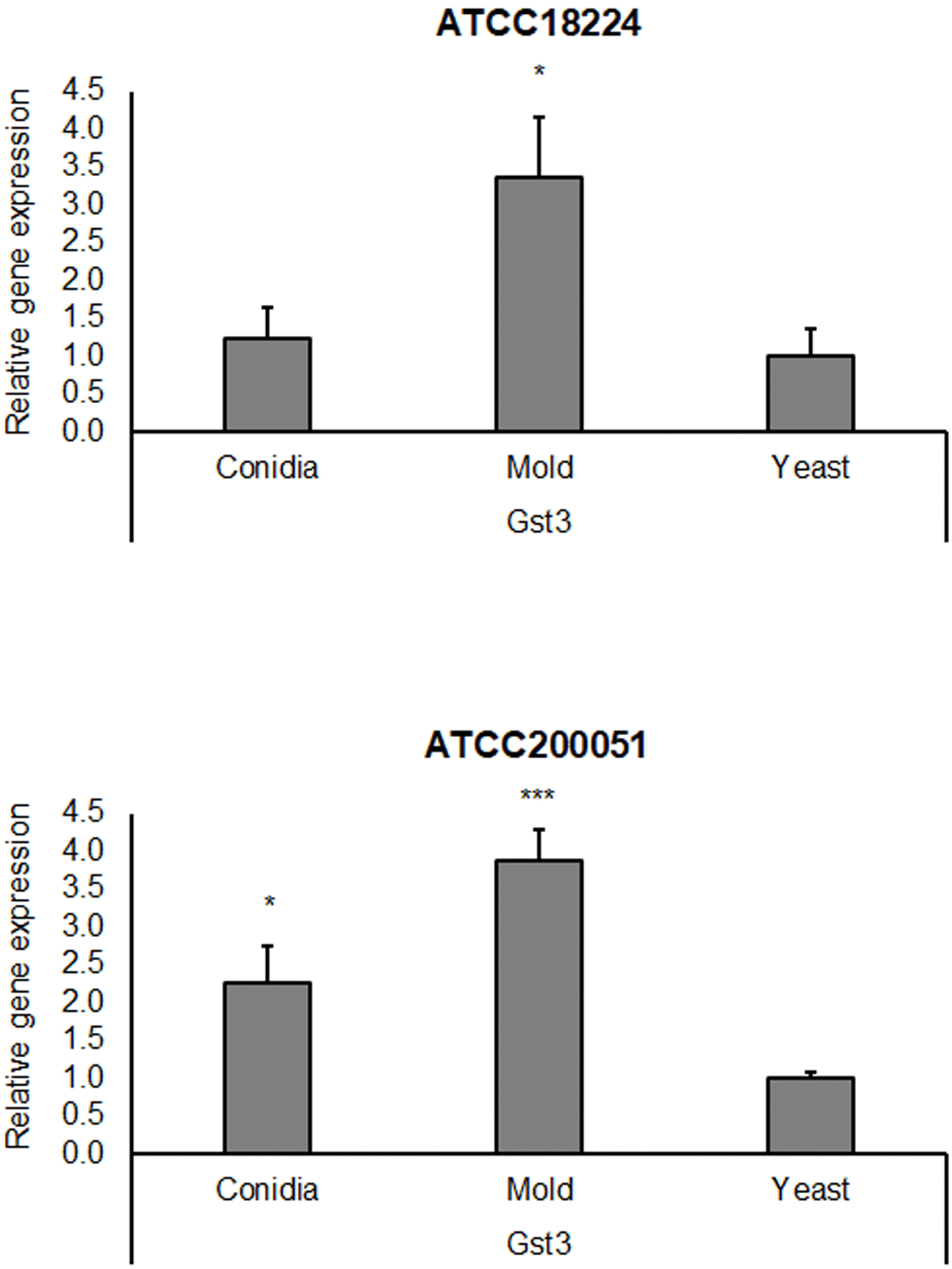

Figure S5. Glutathione S-transferase gene expression profile in *T. marneffei* growing in different cell states. Strains were grown, RNA was prepared, and gene expression was analyzed as described in the legend of Figure 5. Experiments were performed in three biological replicates. Error bars indicate standard deviation. Statistically significant values (* $P \leq 0.05$, ** $P \leq 0.01$, *** $P \leq 0.001$) are indicated.
