# Supplementary material for: Identification of glutathione metabolic genes from a dimorphic fungus Talaromyces marneffei and their gene expression patterns under different environmental conditions
Source: Sci Rep. 2023 Aug 24;13:13888. doi: 10.1038/s41598-023-40932-w (PMC10449922; doi:10.1038/s41598-023-40932-w)
Supplement: Supplementary file 5 — Supplemental Information 5. [file 41598_2023_40932_MOESM5_ESM.doc]

**Fungal species**

>XP_002152894.1 glutathione peroxidase Hyr1, putative [Talaromyces marneffei ATCC 18224] MASATTFYDFSPPDKKGNPYPLTDYKGKVVLVVNTASKCGFTPQFAGLEKLYKSIEAKHPGAFTILGFPC NQFGNQDPGSNDEIQSFCQVNYGVTFPVLGKIDVNGSKAEPLFEWIKSEKPGLLGVKRVLWNFEKALING KGEVVGRWRSITKPESLEATILKEIDIASKDVKGVEVVPTATETAASAAPAEEAKEA

% Ident = 57.96%

>XP_714295.1 peroxiredoxin [Candida albicans SC5314] Hyr1 gpx31 orf19.86

MSQFYELAPKDAKGEPYPFEQLKGKVVLIVNVASKCGFTPQYKGLEELNKKFADQPVQILGFPCNQFGHQ EPGSNEEIGSFCSLNYGVTFPVLDKIEVNGDNTDPVYKYLKSQKSGVLGLTRIKWNFEKFLIDQNGKVIE RFSSLTSPESIGTKIEELLKK

% Ident = 54.78%

>XP_714294.1 Gpx2p [Candida albicans SC5314] orf19.85 gpx32 MSDFYEFAPNDIKGTPYSFKKLQGKVVLIVNVASKCGFTPQYKGLQDLKQKFADQPVEILGFPCNQFGHQ EPGTNEEIEKYCREYFGVTFPVLSKVETNGKNAEPVYKFLKSQKPGLLGLHRIMWNFEKFLIDQDGNVVA RFSSFTKPETIGLRIEEMLKHQA

% Ident = 45.81%

>XP_714296.2 Gpx1p [Candida albicans SC5314] orf19.87 gpx33 MVKSNVESAWETMEDTFSEIQNKFHQIYKPIDTGKPDEDDETTKKESLDITDDSTLSVSPITQLLYLARS KFYDLTPLDNQKSPFPFKNLRGKVVLIVNVASRCGFSFQYNGLEQLNKRFANDDFVLLGVPCNQFLWQEP GTNDQIVTKCKKKYDVSFQILDKINVNGEQADPVYKFLKAQKEGLWGTNRVKWNFEKFLIDKNGRVVERY STFTRPVAIIPKIEQLLES

% Ident = 47.27%

>XP_713880.1 Gpx3p [Candida albicans SC5314] orf19.4436 MGNELLSTARIYTFKIPDAYNNVIDFDQFKNKVILIVNVASLCGFTPQYKELQLLYEKYHERGLEILGFP CNQFGNQEPLQEEEIVESCRRNFGVSFPIMKKTKVNIDCDGHESELYKYLKSEKPGEVGFKGVRWNFEKF IVNRKGEVVARFNSLITPLQLEGFIEQLLSE

% Ident = 77.19%

>XP_754394.1 glutathione peroxidase Hyr1, putative [Aspergillus fumigatus Af293] MVLLPSSLSPPSIIFRCSPLPYISRQSPINRLSSTRAPSLLIRTMASATTFYDFKPADKKGEPFDLASLK GKVVLVVNTASKCGFTPQFKGLENLYQSIKAKHPEDFTILGFPCNQFGSQDPGSNDEIQSFCQVNYGVTF PVLGKLDVNGDNAAPVWTWMKEMMPGLMGLKRVKWNFEKFLISADGKVVGRWASITKPESLEATILKEIE KAKKEGTAASTRKGEGEATAQAKLS

% Ident = 47.06%

>KAH2813746.1 glutathione peroxidase gpx1, partial [Aspergillus fumigatus] DDASFARVWKAQLAQIAIPFMGLPGLTVTTGLVGRVPVGVQVVSGRYREDLCLAAAMSAIYEFSANSLAG EPVPMRRFEGQVMLIVNTASACGFTSQYKGLQELHQKLSPRGLAVLGFPCNQFGGQEPGDAKQIEEFCES NYAVTFPMFAKID

% Ident = 72.94%

>KMP02784.1 glutathione peroxidase [Coccidioides immitis RMSCC 2394] MLPLRRFAYSVAISSAFRRFSTANMSSATSFFDFATPDKKGEPFPLSSLQGKVILVVNTASKCGFTPQLQ GLEALYKKISADYPDKFTVLGFPCNQFGSQDPGTNDEIQSFCQVNYGVTFPILGKVDVNGDKAAPVFEWM KKEMPGLMGLKRVKWNFEKFLISADGKVVGRWASITKPESLEAPILKEIEKMEKGGRTSL

% Ident = 72.94%

>XP_001239998.1 glutathione peroxidase Hyr1 [Coccidioides immitis RS] MSSATSFFDFATPDKKGEPFPLSSLQGKVILVVNTASKCGFTPQLQGLEALYKKISADYPDKFTVLGFPC NQFGSQDPGTNDEIQSFCQVNYGVTFPILGKVDVNGDKAAPVFEWMKKEMPGLMGLKRVKWNFEKFLISA DGKVVGRWASITKPESLEAPILKEIEKMEKGGRTSL

% Ident = 73.26%

>EGC49976.1 glutathione peroxidase [Histoplasma capsulatum H88] MASATSFYDFSPADKKGEPFPLSSLKGKVVLVVNTASKCGFTPQFKGLESLYQSINDAHPGKFAILGFPC NQFASQDPGSNDEIQTFCAVNYGVTFPILAKVDVNGDKTEPLWKWLKSEMPGLMGMKRVKWNFEKFLISA DGKVVNRWSSVTKPESLKAAIEKEIEKSKEVDSPPAPETAAEVPTEPQAQ

% Ident = 67.26%

>XP_010760303.1 uncharacterized protein PADG_04587 [Paracoccidioides brasiliensis Pb18] MSSATSFYEFQPLDKKGEPFLMTGLKGKVVLIVNTASKCGFTPQFKGLESLYTSLSTTYPGKFTIIGFPC NQFGSQEPGTNDEIQSFCSANYGVTFPVLSKVDVNGENAAPLWKWLKSEMPGIMGMKRVKWNFEKFLVSA DGKVVHRWASFTKPESLRHAIEKEIHKAEGQPAEQEPTPEVPTVKSEDQRPEATQPSQPSQL

% Ident = 67.32%

>ODH26765.1 hypothetical protein ACO22_04450, partial [Paracoccidioides brasiliensis] KGEPFLMTGLKGKVVLIVNTASKCGFTPQFKGLESLYTSLSTTYPGKFTIIGFPCNQFGSQEPGTNDEIQ SFCSANYGVTFPVLSKVDVNGENAAPLWKWLKSEMPGIMGMKRVKWNFEKFLVSADGKVVHRWASFTKPE SLRHAIEKEIHKAEGQPAEQEPTPEVPTVKSEDQRPEATQPSQPSQL

% Ident = 62.58%

>XP_016585654.1 glutathione peroxidase [Sporothrix schenckii 1099-18] MAGKTFYDFKPLDKTGKELDLHKFEGKVVLIVNTASKCGFTPQFAGLEKLYKSLRASYGDDVEFLGFPCN QFGAQEPGSNDDIQSFCQINYGVSFPILSKIDVNGDNAAPLYNWLKDSKSGLLGLKRIKWNFEKFLVGRD GAVIERWASTTKPEALEKPIVDALGGKPTAAPTKTEEPAAAPAATATA

% Ident = 50% (gpx1)

>XP_570772.1 glutathione peroxidase, putative [Cryptococcus neoformans var. neoformans JEC21] MTFFDSIASKFGYESLPGDVANKSFYDLKAKLPGSKGDLDFSTLKGKVVLIVNTASKCGFTPQYTGLEEL HKTYGDKGLVVLGFPSNEFGGQEPGSDDDIAQFCTLNHGVTFPLMKKSEVNGKNMNEVFAWLKSQKGENV GGLAGTTAIKWNFTKFLINKEGKCVGRYGSSTKPEKLKEEIEKLL

% Ident = 44.76% (gpx2)

>XP_568531.1 glutathione peroxidase, putative [Cryptococcus neoformans var. neoformans JEC21] MSDIYSYSVEFPKSTLPLSDLKGKTLLFVNVASKCGLTPQYKDLQALHEKYGDKGLAIIGFPCNQFKAQE PGTDDEVLQFCQVNYGVTFPIAKKGDVNGENTQPIWKYLKENAEPPVSDIDWNFSKFLVKDGKITWFPAR STKVSDVEAAL

% Ident = 55.76%

>NP_012303.1 peroxiredoxin HYR1 [Saccharomyces cerevisiae S288C] MSEFYKLAPVDKKGQPFPFDQLKGKVVLIVNVASKCGFTPQYKELEALYKRYKDEGFTIIGFPCNQFGHQ EPGSDEEIAQFCQLNYGVTFPIMKKIDVNGGNEDPVYKFLKSQKSGMLGLRGIKWNFEKFLVDKKGKVYE RYSSLTKPSSLSETIEELLKEVE

% Ident = 45.28%

>NP_012899.3 glutathione peroxidase GPX1 [Saccharomyces cerevisiae S288C] MQEFYSFSPIDENGNPFPFNSLRNKVVLIVNVASHCAFTPQYKELEYLYEKYKSHGLVIVAFPCGQFGNQ EFEKDKEINKFCQDKYGVTFPILHKIRCNGQKQDPVYKFLKNSVSGKSGIKMIKWNFEKFVVDRNGKVVK RFSCMTRPLELCPIIEELLNQPPEEQI

% Ident = 56.96%

>NP_009803.3 glutathione peroxidase GPX2 [Saccharomyces cerevisiae S288C] MTTSFYDLECKDKKGESFKFDQLKGKVVLIVNVASKCGFTPQYKELEELYKKYQDKGFVILGFPCNQFGK QEPGSDEQITEFCQLNYGVTFPIMKKIDVNGSNADSVYNYLKSQKAGLLGFKGIKWNFEKFLVDSNGKVV QRFSSLTKPSSLDQEIQSLLSK
