# Supplementary material for: Identification of glutathione metabolic genes from a dimorphic fungus Talaromyces marneffei and their gene expression patterns under different environmental conditions
Source: Sci Rep. 2023 Aug 24;13:13888. doi: 10.1038/s41598-023-40932-w (PMC10449922; doi:10.1038/s41598-023-40932-w)
Supplement: Supplementary file 6 — Supplemental Information 6. [file 41598_2023_40932_MOESM6_ESM.docx]

**Supplemental data S2. List of 48 GST protein sequences used in phylogenetic tree construction.**

>Tm Gst1 XP_002151959.1

MDGLVIKCTSFSPLTRWLFFNQCSLFTSIDNSCTLLRSNIYSSFSIPNKLNMVQTLELWGHWGAPNPWKV

AMVLEELSLPYQINYLELTDVKTESYLEVTPNGRLPALRDPNTGITLWESGAIILYLVDQYDRDGKISYK

DSPEKYLCQQWLAFQISGQGPYFGQATWFARFHPEKIQSAIDRYINEILRVISVIDLGLRRNPAAGNWLV

GDKCTYADLSFATWALVGYGLLKELGKNEGLEKDYPNYTQWIAKMEEQESVKKIRDVMNQGRIAHSLKV

>Tm Gst2 XP_002148492.1

MTTNILPIKLYGGVLGPNPLKVGLILTALKLPFESIIIPMDQLKKPDYEALNPNGRLPTIHDPNTNFTIW

ESGAIIEYLIERYDTDEPRKFSFPPGSAEAQLARSFLYLQTTGQGPYYGQAYWFKNFHHEKIPSNVERYV

TEIKRVTGVLDKWLGEQKEAHKDNIQDGPWLVGNKFSFADVAFIPWQKIALANHAEDGFDFNTYPHAKDW

FERMAAKESLKAVLDTYDVEMAKRRQAKK

>Tm Gst3 XP_002144011.1

MAAHRTPDITLYTAQTPNGIKISIALEELGLPYKVKKLEFSKNEQKEPWFLEINPNGRIPAITDTFTDGK

QISIFESGSILQYLADNYDTEYKISYPRGTREYYETANWLHFQMAGVGPMQGQANHFTRYAPEHIEYGIN

RYQNETRRLYGVLDKHLSTSKSGYLVGDHISIADISHWGWIAAAGWAGIDIEAFPHLKAWEEKLAAREGV

EKGRHVPEKHTVKEVLKDKALVAEHSASTLQWVQKGMADDAKK

>Tm Gst4 XP_002149413.1

MSTPKITLYTSHLCPWAHRAHIALKELGLPFEEVLIDLSVPREPWYLEINPRGQVPALVYDGNIITESAI

VARFLADAHPSHLLPPSTGIENALYRARLDWFVDAFISKVNPHLFGSARAATEEDRDKSAELLVAAVAKD

IEPRLVEGKGPFYGGSETLTFAEV

>Tm Gst5 XP_002147066.1

MRAPPELKAIHPLGKSPIVTVERPEYAQPLVLAESGAIVEYLLNHFGGEEKGLVPRKYASEEDAAKNLEM

EEWLRYRFYMHYVEGSLMPQMVTGLILDNIRNVPIPFFLKPIPRLVADNIQMNWLDKQFAVHFPFLEGQL

KSAPGTSDGQRGGLCGAKFNAADILMSFPIIAATGRGLITKESYPEIVAFAERLKNDDGYQRAVKKIEEV

DGKFVASL

>Tm Gst6 XP_002152077.1

MELSIPPNTRALVILPNIERLPKHVASDEDEGNWVGSGHHKFSVPFRWRDYSSEWPPKPLIPIMRRSEPD

SIAKIADTTMQKLPPPTLHHLSSSSSLRVLWALEELFLSSGLEYNLKNYKRVKGRAPEDLKTVFPLGKSP

VLEIPGVNLFRPLPFLHDDSSNNNEIKTIMTESRLILQLLSDKYSNGEWVPETAEDKERDSYFLEFANSS

LTGVVNSILYFEIIPTMSPWLVRPLMSAIFNPIAKILKQGLDPHFDLMERALSDEKPWFSGSKIGLADFT

LTFPMDTAVQRQFLDEKKYPKLAGWVKRVHDRPAYQNALKKGGSYDLIRYDN

>Tm Gst7 XP_002147180.1

MLTVHHLECSQSERIVWLCEELAISYELKRYNRSPIFAPPELKALTPMGGAPIITDSTFDPSNPLTIGES

AAIVEYIVHKHGGGRLILSPSDKNYSDYLYWFHQSNTNLQGAIFRALLVGQLRLAPENPIQKGVDAKVKA

ILNMLNDRLNQVTWLAGDEFTVADIMTVTSVTTMRFYYPIDLTGYDGILLWLQRVGERPAYRSARQKGDP

GLVPCLGAATPELFPAWAAISQ

>Tm Gst8 XP_002145828.1

MTSFTLYGARGSTCTDRVRLTLAEGGFTDYELVFLDLQKGMQRSQEHLKRHPWGKVPAITFPDGFTLYES

RAICKYLARKYSFPLLPPDSDIETAALFDQAQCVEMSYFAEPAGKIAFEKFVKRFLGLIPNEAVISDALR

SLEMFFDVAESLLHDREYMAGNDFTLVDIYYIPLIQRLFTCGYGDIIVSRKAVNAWWERCVNRPAIQRMW

AADKEAAV

>Tm Gst9 XP_002147102.1

MGIVIHGASKATCTQRILATLIEKGVTDYELKTISTRAGEHKQPEYLKKQPFGVIPVLEDGDFLMHESRA

ICYYIASKYIDQGPKLIPDVTDLTGNATFQKWANSYASPITSEKLFKVMRGGSPDPKLVEHYEAGFLPKL

DVFEGILSKQKYMGGDEFSLIDVYYLPYTQKLSEVGCGHFITDRPHVKAWWERVSARDSWQKVLALP

>Tm Gst10 XP_002145896.1

MSVVAEPRGFIDFYNKPGSSNGAKIAIILNELELTYRHHEIRSNPTSPLDKDTYHAISPKGHFPVVTDIH

PNGFKVSLDQTGAIAQYLVNEYDQEDHTISFPRRSAEDIEAMNWFFFGATRVASSHDEAVHYKKDAPENT

YSVDRFRSRTTGLFFALEQRLKETGDYLVGHKFSIADIAQVPFVVAAEEAGISIEIFPALTAWYNRIMSRPGVRKGLLVAGIEFSA

>Tm Gst11 XP_002148730.1

MATPIKFYYSPGSCSFAVHILLFEANVSFEPIRVEMGKFPKEFLALNPKGRVPVIILKGDHNNDQVITEA PAILTTVSLQVPERKFLGSNNLETARVYEWMNWLSGTLHGQAFAGFWRPDRFMNEPTEEDARRIAEKAVE TILSCYETIESKLYSSYTASGAPFAVGNAMTVVDPYLAVFYRWGVRNLKLDMTRYQKYTALWKELEKLDS FRLALGQQAGSWE

>Tm Gst12 XP_002146237.1

MTSQTRVNAAAPAGTEHETEPPQPATPSVVQPKKLFGVPAPVKRIFDKFPLTTYEAEGIPGNVHTSGSSE RNRLFVFIDPKQATAGAPSFNPQCLRWQAYLKFVGIDFDLIPSNNHASPTGALPFLYPALPAATRSPIPS NKLQKWAIEQVHCEEEQQLNIRFDVYASLIDHRIRNAWLHQLYLNELNFNHVARKRYIDPATSSSAVQTA LAVQLQQAARDELLKYSEYIDVNTLEADADSAFEALSILLGDNKYFFNRNQPGLFDANVFAYTHLILDEK MGWRYNRLAHSLSKYENLLRHREALLEKYFA

>Tm Gst13 XP_002153321.1

MSFGKLYGSTGHGRTIPSVVAAKANGLDLEVVETAPGKASTDINPLGKVPAFVGANGFKLTEAMAIAIYI TSQNEKTTLLGTTKQDYASILRWMSFANTDLITRLASWYLPLVGVLPYNKKTVDEQATASAAALKVLETH LTANTFLVGERITLADIFTAGIIRRGFATVLDKKFRSEFVAVTRWFNTVVNQSYWKDVIPEATLVEEAIK YTPPKKEEKPKKEAPKPAAKEEDDEEPDVKPEPKAKHPLEALGKPTLILDEWKRQYSNNDTRPVAMPWFW QNYKPEEYSLWLVDYKYNDELKLTFMANNLIGGFFARLEASRKFLFGAASVYGENYKCLIRGAFLVRGQE CLPAFDVAPDWESYEFKKLDHTKEEDRKLIEDLWAWDTPVVREGKEWPHVDGHVFK

>Tm Gst14 XP_002143473.1

MITVPGSWFYLSRNLDLVTKEEALSTTMSSASSRPVLHYLELGTLGRGEVVRLFLNDAGIDFEDIRYAYD HTWAATSAELQAKGLTVTGKVPVLEFNGTILTQHIPILRYLARELNDYDGQTSLEKWVVDAVSDIYIDWR TQYVANLENASDSFKNKYTPEYYNILSHYYSQRGGPFLLGDRATYADFVIYQSLDDDRKTSTLPETLPGA IVAFKEAFESRPRVKAYLESRRNMNINK

>Tm Gst15 XP_002144827.1

MDGHHVSSDRVTLYVKKATHTSGPNVAKLLILLDLLHIPYDYKVLTSPSESEWFGEINPYKMVPAITSLE ESATAVLNVFESSACLTFLADKYDREGLYKGKTLAERARVSSWLMAYTAGLGPTGKWWLTLKVGHASTIG NALEILAAAIKKEYSILEARLNEPGQQYIALADRLTIADIAILPFANAQIAMSAQIEFGEYPALKAWSEK VLAMPEVGRAFMRVQTFGHEDEAKEER

>Tm Gst16 XP_002146532.1

MVSITVPDNYGYVFCFFLSRLPQVHLHLERISRFHFNPVQPTRIAKLIIYSAVIAVALGGIPLLSFVQGV

VVTSLRKPAKVRYPQCYATPEQCKENPAAQKFNCAQRSHGNLLENMTQTMLFMLVAGLKYPNATAALGTA

WIVFRALFAHGYITSEKANGGGRYNGGMFWLVQGALWGLAVFGVGLELLKF

>Tm Gst17 XP_002150784.1

MGGLIHAYYDCVSPYSFFAFTHLQEVRAVLASHGVSIETHPIFLGGIMNGSGNQPPWLVPAKAKLGQLEL

ERAIKYWKVEPFKIPKIFPILTILPQRALTYIKHTYPTAQFENTAHLYWQWFLYKHQDISKPDILRELLA

SPEAGFSREQAEEILTAAMTDKKWKEELTATTQAALDKGAFGAPFFWVVKTDGHGRTVAEEPFFGSDRFH

QMWEYLGLPYEDLKLLPRVEAKL

>Tm Gst18 XP_002151334.1

MADQQKQQTQPKTYHKKATGEAQSTVKKRSQENDLKLFGSCFCPFVQRVWIALEAKGIPYQYIEVDPYDK

PPELLAINPRGLIPALLHGNWGCYESTVLLEYLDDLDEGTPLLPPGDARLRAHCRLWGDHINRNIVPSFY

KVLMEQTPQMQAKHAAELQEDIEKLVNASHVHGPFFLGPSMSYVDIQLAPWIIRLSRVLKPYRGWTEPTI

GSRLGRWIQAIEGNEHVIATTSNDDLYLESYQRYSKNRPNTSQLANAINSDRGLP

>Tm Gst19 XP_002143279.1

MSASEGKPVLHYLDIGSLGRGEVIRLFLKDAGIDFEDTRYPFNDSWKDTKAELQKKGISRTGKVPVLEYK

GIYLSQHIPILRYLARELKAYDGATSLEKHLVDVVADLYIDWRAQWVANLEKLSDSYKNEILPDHYKTLA

YYYGKNGGPFLLGDRVTYADFAVFQSIDNNEKIGADATLPEELLKFKTAFEARPRVAAYLKSGRNTKA

>Tm Gst20 XP_002147405

MANITVYRGFPTTPKCVWSPFVNKLEARLRFAGIAYNLEQGSLLKAPRGKIPYISIRAGIFGGYAAHQLT

ESGILPDLNEHLSPAEKTMDQALRALLEDKLYFYNGNERWNENYYTMRDGVMASIPYPIRVIVGLLAWRN

NNAGLYSQGTGRFSAEEIHSFRDKIWHSLDDLLAESRHKAPSGQKVFWALGGKGPTEADTSLFAFVIAGL

VCDAGPDSRKLIRTLPNVIDYARRIHEEYFADYEAPAW

>Sc URE2

MMNNNGNQVSNLSNALRQVNIGNRNSNTTTDQSNINFEFSTGVNNNNNNNSSSNNNNVQN

NNSGRNGSQNNDNENNIKNTLEQHRQQQQAFSDMSHVEYSRITKFFQEQPLEGYTLFSHR

SAPNGFKVAIVLSELGFHYNTIFLDFNLGEHRAPEFVSVNPNARVPALIDHGMDNLSIWE

SGAILLHLVNKYYKETGNPLLWSDDLADQSQINAWLFFQTSGHAPMIGQALHFRYFHSQK

IASAVERYTDEVRRVYGVVEMALAERREALVMELDTENAAAYSAGTTPMSQSRFFDYPVW

LVGDKLTIADLAFVPWNNVVDRIGINIKIEFPEVYKWTKHMMRRPAVIKALRGE

>Sc GTT1

MSLPIIKVHWLDHSRAFRLLWLLDHLNLEYEIVPYKRDANFRAPPELKKIHPLGRSPLLE

VQDRETGKKKILAESGFIFQYVLQHFDHSHVLMSEDADIADQINYYLFYVEGSLQPPLMI

EFILSKVKDSGMPFPISYLARKVADKISQAYSSGEVKNQFDFVEGEISKNNGYLVDGKLS

GADILMSFPLQMAFERKFAAPEDYPAISKWLKTITSEESYAASKEKARALGSNF

>Sc GTT2

MNGRGFLIYNGGEKMKQKMIIYDTPAGPYPARVRIALAEKNMLSSVQFVRINLWKGEHKK

PEFLAKNYSGTVPVLELDDGTLIAECTAITEYIDALDGTPTLTGKTPLEKGVIHMMNKRA

ELELLDPVSVYFHHATPGLGPEVELYQNKEWGLRQRDKALHGMHYFDTVLRERPYVAGDS

FSMADITVIAGLIFAAIVKLQVPEECEALRAWYKRMQQRPSVKKLLEIRSKSS

>Sc GTT3

MPTKSTFSRWKKADLIDLANKLEIDGFPNYAKKSDMIDYLESHLNHLEKPVDFKDDYPEL

RSFYESMTVDQSKDERNEYGSGSGNGSGSGSCDTATNDSDLEKAYIKEDDDEKPQSGDET

SATKPLSSRNANSNAKTNFNLLDFSTDNDSSTSAFTKFKFNFQEYLSDIRYQTQKLNENV

QDYLSTISAVDTIFSLLEFSFLVRNILAAGQPTSSSSLASSLEAAVAAHNKYQYTLDFCL

PILTWLLFFRGIPTLVSYYINFIRYDLNIELDPMTFNLTKFLISLAIFKTCNNKNIDFHS

FRCVNQLWTQLCTVNRSLGMVPLVFSMVSCLLTLYVL

>Sc SAM35

MVSSFSVPMPVKRIFDTFPLQTYAAQTDKDEAVALEIQRRSYTFTERGGGSSELTVEGTY

KLGVYNVFLEANTGAALATDPWCLFVQLALCQKNGLVLPTHSQEQTPSHTCNHEMLVLSR

LSNPDEALPILVEGYKKRIIRSTVAISEIMRSRILDDAEQLMYYTLLDTVLYDCWITQII

FCASDAQFMELYSCQKLSGSIVTPLDVENSLLQKLSAKSLKISLTKRNKFQFRHREIVKS

MQGVYHNHHNSVNQEQVLNVLFENSKQVLLGLKDMLKSDGQPTYLHLKIASYILCITNVK

EPIKLKTFVENECKELVQFAQDTLKNFVQ

>Nc EF1Bγ XP_961215.1

MAFGKLYTYEANPRSTAILAVAKANNLDLEVIKVDLEAAIEEYKKVNPLGKVPTFVGADG YTLFECIAIAIYVASQNEKTTLLGKTKQDYASILKWLSFFNTEVLPPLAGWYRPLLGKAP YNKKAVEDAQATALKAISVAEAHLKNNTFLVGERITLADLFATGIIARGFEFFFDKAWRE QYPNVTRWYTTVYNQPIYSAVAPPFALLDTPKLTNVAPKKAEAPKPAAPKPAAAPAAAAE EPAEAPKPKHPLEALPRASFPLDEWKRQYSNVDTPEALKWFWENVPFTEYSIWKVNYKYN DELTLTFMSNNLIGGFNNRLEASRKYLFGCASVYGTNNDSVIQGAFVIRGDDWKPVFDVA PDYESYEFTKLDPQNPEDRAFVEAEWSWDKPALVNGKEYPHASGKVFK

>An EF1Bγ XP_664167.1

MAFGKLYGRPDNTRTIAVLVAAKHNDLELELVETQANPAADFNKSDAYTKIQPLGKIPAF EGANGFTLSEVIAIAVYVTSQNEKTTLLGKTKQDYASILRWLSFANSELLVSFGSWFRPL LGLDPYNKKNVEDASKAALKKLGVLNTHLTANTYLVGERITLADLFTASLLTRAFATVID KKVRADYPAVTRWYQTIIDQSAFKAVVENPVLIDEAIKYTPPKKEEKPKKEAAPAAAAPA AEEDKPAPKPKHPLEALGKPTLILDDWKRTYSNEDTRSVAMPWFWQNYKPEEYSLWKVNY KYDNELKLTFMANNLIGGFHARLEASRKYLFGCQGVYGENYACVNRGVFLVRGQEALPAF DVAPDYESYEFIKLDHTNEADRKYVEDIWAWDTPVVVDGKELPNVDGHVFK

>An EF1Bγ XP_681076.1

MPFGTLYTRPFNPRSLAILAIAKANNLPLKIKTITSFKDATEEYLQLNPLGKIPTFVGAD GYVLTESIAIALYDSNTTLLGTTGQEYASIIRWMAFGITEILPALGGWFNPLIGRANFNA DNIYQSKDDTLARLKILDNHLCGREYLVGETLSLADLFVLGIVQGAFRFFLDKRWRDEHR NLSTWFERVHALPIVVDVAGPPVLAEYEMPIQPPK

>An EF1Bγ XP_659199.1

MNTLNAPRNPTRHPAMTADTLVDAPALPHQNGSTEEKLKERGSFGKLYTYKRSPRALGIQ AVAKSIGLELEQVELQPANGVPDFYWNLNPLGKTPTFVGADGLVLTECMAIALHVTNEDS TTTLLGSSSLDFVQIIRWISFTNTDVVTRMASWVRPLIGYTPYSKEEVLKAQQQTTQAIG VFEDSLRDRKYLVGDRLTLADIMCVSLVSFGFAQIFDKEWREAFPYFSGWYMMVMHLPIM KAVVEEVPFVEEGLPNAPPTEPFRAP

>Sp EF1Bγ NP_587885.1

MFLGTLYSFKTNTRTVCLLELAKLLDLQVDLVETYPHKFSADLAAKFPLQKLPVFIGADG FELSEVIAIVKYFYEKGKHNDKEGLGPVNEVEEAEMLKWMCFINFDIVTPQNVRPWVGMF RGNIPYEEKPFKESATRAIDSLKIPNELVKDRTYLVGDRFTLADLFFGSLLRIFFNSIID EKTRKELPHLTRYYITMFHQAKLETYFPLELPLTVTVAKK

>Sp EF1Bγ P40921.1

MSVGTVYGKIGSPRVLFCVSVAAVAGVEVEHVDVQPHNFPADLAAKFPLQKMPVFVGKDG FPLSETLAIAFYLASLNKTRALNGTTAEEKAKVLQYCSFTNSELPGAFRPIIAPRVFGAP YDEQAAKEAETAIALIFARFDEELASKTYLVGSRLTLADIFFTCFLKFGATYVLTKSYLA KYTHIYRYYQTIYHQAKLDAITEPLKFIDQPLPIIKAENKEAAPAKKAEKKKDEKKKNAP KPQAERPAKPPKHPLASAPNGSFDIEEYKRVYSNQDTRSGALPWFFEHFDPENYSVWKVD YSYPEDLKQPVFMTNNLIGGFFQRLEASRKYIFGCCVVIGENGDNTITGAFVIKGHDYVP AFDVAPDWGSYTFTKLDINKPEDKAFIEDAWAWDKPIEGREVADGKVCK

>Sc EF1Bγ NP_012842.1

MSQGTLYINRSPRNYASEALISYFKLDVKIVDLEQSSEFASLFPLKQAPAFLGPKGLKLT EALAIQFYLANQVADEKERARLLGSDVIEKSQILRWASLANSDVMSNIARPFLSFKGLIP YNKKDVDACFVKIDNLAAVFDARLRDYTFVATENISLGDLHAAGSWAFGLATILGPEWRA KHPHLMRWFNTVAASPIVKTPFAEVKLAEKALTYTPPKKQKAEKPKAEKSKAEKKKDEAK PADDAAPAKKPKHPLEALGKSTFVLDDWKRKYSNDDTRPVALPWFWEHYNPEEYSIWKVG YKYNDELTLTFMSNNLVGGFFNRLSASTKYMFGCLVVYGENNNNGIVGAVMVRGQDFAPA FDVAPDWESYEYTKLDPTKEEDKEFVNNMWAWDKPVVVNGEDKEIVDGKVLK

>Ag EF1Bγ NP_984243.2

MSQGTLYVKKTCRSMLPQSIVEHYNLDVSIVDADKNEEFEKKFPLKRAPAFSCAAGNLTE TMAITYYLVNLIQDEKAKAALLGSTLEEQAQVLRWESLTNTNFIDDVASAFLYLEGIVPF NKRDMENRIASAERIAEVYEQRLRNYTYLATENVSVADILAVGGFELAFTNIWGAQWRAA HPAICRWFNTLANSPLLKKHIDLSKLVQEPKKVAAPKKEKKEKKEQPKKKEQPKKEKEAA PAAEEPAEPKKPKHPLSLLPPATLNLEDWKRKYSNEDTRPVALPWFWERYNPEEYSIWKV GYKYNDELTLTFMSNNLIGGFFNRLSASTKYMFGCLVVYGENNNNGIVGAIMVRGQDAIP AFDVAPDWESYDYVKLDTSKEEDREFINDMWAWDKPVMVDGVAREIADGKVLK

>Sp GSTN_2 NP_588171.1

MSNTHITDWSSKDGEFRRQVSSFRERISPEHKYFQPEKDRYHLYVSYACPWAHRTLIVRK LKGLENVIPVHVVGWLMGPNGWNFDKENDSTGDPLYNSPYLRNLYFRADPNYNMRFTVPV LWDSKYNTIVNNESAEIIRMFNDAFNEVIEDEEKRVVDLYPSSLRTKIDELNDYFYDTVN NGVYKTGFATTAEAYEKNVRVVFQGLDRLEQVLKESKGPFLLGDHLTETDVRLYTTIVRF DPVYVQHFKCNIGTIRHNYPHINQWLKRLYWKHPAFHETTDFKHIKCHYTQSHTQINPLG ITPLGPIPNVEYF

>An Zeta XP_659499.1

MSTNSDLRVTLYTYFRSSCSARLRIALALRSISYTSVPINLLKGEQSSTKNTAVNPSATV PTLIIEHVDRSQSPITITQSLAALEYLDEAFPDNPNPLLPPISNPQQRALVRSLASIIAC DIQPVTNLRILQRVAPFGVDRAAWSKDLIEAGFAAYEAIARDSAGVFSVGDTITMADVCL IPAVWGAERAGVNLGQYPTIKRVAEALEKENAVKEGHWRTQQDTPTEFRC

>Ce Zeta NP_509962.1

MSNQKPVLYSYWRSSCSWRVRIALALKNVDYEYKTVDLLSEEAKSKLKEINPAAKVPTFV VDGQVITESLAIIEYLEETHPDVPLLPKDPIKRAHARAISLLVASGIQPLHNLKVLQLLN KKEAGFGGQFAKQFVVEGLTALEILLKQHSGKYAVGDDVTIADLSIPPLIYSANRFNLDL SPYPTVNRINETLADIPAFIAAHPDNQPDTGLNA

>Hs Zeta AAB96392.1

MQAGKPILYSYFRSSCSWRVRIALALKGIDYKTVPINLIKDRGQQFSKDFQALNPMKQVP TLKIDGITIHQSLAIIEYLEEMRPTPRLLPQDPKKRASVRMISDLIAGGIQPLQNLSVLK QVGEEMQLTWAQNAITCGFNALEQILQSTAGIYCVGDEVTMADLCLVPQVANAERFKVDL TPYPTISSINKRLLVLEAFQVSHPCRQPDTPTELRA

>Ts Theta XP_002340542.1

MAFKLYGSPLSGCTQRVLLVLAEKGVEDFELLPVNLMKGEHKMPNYTEKHPFGVIPLLEE GEFRLFESRAISRYLAIKYKDKGTSLVPSAGDWAGWALFEQWAAVESSNFHYYCEQILTQ KMWNPYKGLPTVDAILDDATKRFEEKLDTFDKVLGTQEYLGGKEFSLIDIFYMPAVALLF RAGAGSLIELRPNWKAWWQRVSTRPSWEKVSASAAAAAAAMAGNK

>Ts Theta XP_002482063.1

MPAFTLYGSRGSTNTDRVRLTLAEGGFTDYELVLLNLSKGEQRSKEHLKRHPWGKIPAVT SDEGFTLYESRAICKYLAKTYSFRLLPPNSDVEATALFDEAQSVEMLYFAEPAGRIAFEK FAKRFMGLPPNEAVIADALRSVEMFFDVADRLLHNRDYMAGNEFTLVDIYYIPLIQRLFA CGYGDVIDSREAVRAWWDRCVKRPAIQRVLVADKEAAVAASE

>An Ure2CBF76508.1

MSGVTPALRLLKGCFSQFSFNSNLRIPLKHSQLLNSASSRPYNRLFSTTSPVMSRPDITL YTAQTPNGIKISIALEELGIPYKVEKIDISKNVQKEPWFLEINPNGRIPALTDTFTDGQK IRLFESGSILTYLAEQYDKDYKISYPRGTREYYETISWLYFQNAGVGPMQGQANHFSRYA PERIEYGVNRYVNETRRLYGVLDKHLANSKSGYLVGDHITIADISHWGWVAAAGWAGVDI DEFPHLKAWEERLAAREGVEKGRHVPSPHTIKDLLKDKKKAEEIAAQGRAWVQEGMKNDA AK

> Pc Ure2 XP_002561977.1

MSSNITLYSWPTPNGVKASITLEELGLSYKAEGLDISSSSNPQKEEWFLKINPNGRIPAL LDGSQRVFESGAIMTYLVDKYDTDRKISYAPGIPEHAEQTSWLMFQMAGLGPIQGQANHF RLFANTRSDYAIKRFVDETRRLYSVLESRLNESPYLAGEKYTIADIASFSWVRGSPISLE IDLSEFPALKKWVDEIDKRAAVQRGLDIPHSTWTPEQKAEIFRNCRAKIDAMTTSDQH

>Sp Ure2 NP_588298.1

MAQFTLWSHAHGPNPWKVVQALKELDLTYETRYVNFSKNEQKSPEHLALNPNGRVPTLID HHNNDYTIWESDAILIYLADKYDTERKISLPRDHPEYYKVIQYLFFQASGQGIIWGQAGW FSVYHQELVISAITRYRNEIKRVLGVLEDILKDRDYLVANRFTIADLSFISWNNFLEIIF AEGKFSIEEEVPQLDFEKEFPRTYSWHQRLLARPASKATFEERSKALDN

>Sp Ure2 NP_588517.1

MAHFTLYSHAGGPNPWKVVLALKELNLSYEQIFYDFQKGEQKCKEHLALNPNGRVPTLVD HKNNDYTIWESDAILIYLADKYDTDRKISLSFDDPEYYKLIQYLFFQASGQGVIWGQAGW FNFFHHEPVVSAVTRYRNEIKRVLGVLEDILKDRDYLVANKYTIADLSFIPWNYNLGGLF GEGKFSFKEEVPQLDFEKEFPKAYAWNQRLLARPAVKATFEELAKAKEQH

>Mo Ure2 EAQ71536.1

MSSSIKPIVVHGKIGPNPPKVHMLLNELGLPHTTTPHDFTSIKQEPYLTKVNPNGRMPAI EDPNTDLTLWESGAILQYLVETYDKEHKVSFPAGSNESHLAKQWLFFQTTGQGPYYGQFV WFTKYHEPKVPSAVERYAKEINRVTAVLETHLSKQADDADGNRWLVGRRFSYADLAFVPW QYYAGMLAKDYYKSDDYPHVKKWLDALVARPAIKKVVEEDMAR

>Ani Ure2 CAK39793.1

MQTTLGRALSKMTKPLLLHAHATGPNPIKIAIALEALQVPYEVQQWEFGDDPQKGVKGEA YLKINENGRLPSLQDPNTGVVAWESGACMNYVRRVYDRDGQLGPVGKTEQDIVDLEKWEY FLLTNLAPMLGQVNWFRNYHAVKNQDALNRYTEQAYRCFGVLEGQLKKSNGESVLPGKIT AVDYHSEPWVRQYSFAGLSLDSYPLIQRWLAGMAGREEVKRAYVEIKGKGPE

>Pb GST N_3 EEH45625.1

MENPEAQLQHSLRSLAATEDIRSLPTDPSLTSRISAHFEAAAAAAAATGGPQPFAVLQAT TTGTTAAGTHLQHSPHAERLIDNSVNPPSLPPHQQQLSHAHHQHQFHTPNNNGFLDHRNN GQVDMLQQLQTPLPAHLLNQFHSTSPNIEAANHSDQSPSPASAHNRGEGHFKNMKLIPNP PDLKRWRERLFNVDDDPIVMSEEEFQTYFPHVDNVYSHRSTQKYKRKLFVSHYWDCRLKG RPPGTPKSRDPNKKKRKRTARERDLCDVKIKITEYFPGALAVPPNLLGLDAMSSLEQNMQ NAAAAVATGTDFLDPVVAGQNELSRVANSNNPQPQAEPHYQFGVLTLRKTLPQGHPGYTG EKYYTIQRVNGNGGNGRADGIGGPHRHTLEESDRVKKNSVQRHMLKVEKERRKTMAHQKT YHKKASGLAATTVKKHAKEADLKLFGSCFCPFVQRVWIALEVKGIPYQYIEIDPYKKPDS LLEMNPRGLVPAIRHGNWECYESTVLLEYLDDLDTGNLLLPSRDPQLRAHCRLWADHINR HIIPTFYRLLQEQDLQKQIQHSEELKEEISKLVNASHMHGPFFLGPTISFVDIQFAPWIL RLSRVLKPYRGWPDPEKGSRWAAWVDAIEADERVRATTSADDLYLDSYERYAGK

>Cp Metaxin 1EFW15805.1

MVLELHTWGPAFGLPSIDAQCLATIAYFALALPTNGSPEWVLVPGSDPKIVPTNELPAVW TGSRWISGFRNIVAFLKQYSDGEWDLDRWMGPGEQADCVAFSSFLELHGQSLIDLSLYVS SDNYTSVTSPAYGTLLQWPNQWIIPPQVRSQAKARTEHLGLSSLDLDAVEEERQQGRDIN SVAAGQIPKSLATRPRQTVSGLLGKPSQKSRIRLEGLTASFVEPLQEMLEKKGYLLSDNI PSSLDCLALGYLSLAMVPELPFPWLKDDIQAQAPRLGAYVKKLGGRCFGGSVDAAAVLSG IQAGTGSRLPWQIPERISLGGIGLRIFEGIADSIPVVKDIRLSRRLKQMGQDKSLESEEV LALADGYKREALTSAATVALGLGMFMVYLFTAGPLQVSFGTEDDHSSSSAENEEAGGDDG EEKGEKGSSGKPFDLGEVGSMLGL

>Cp Metaxin XP_003065220.1

MVLELHTWGPAFGLPSIDAQCLATIAYFALALPTNGSPEWVLVPDSDPKIVPTNELPAVW TGSRWISGFRNIVAFLKQYSDGEWDLDRWMGPGEQADCVAFSSFLELHGQSLIDLSLYVS SDNYTSVTSPAYGTLLQWPNQWIIPPQVRSQAKARTEHLGLSSLDLDAVEEERQQGRDIN SVAAGQIPKSLATRPRQTVSGLLGKPSQKSRIRLEGLTASFVEPLQEMLEKKGYLLSDNI PSSLDCLALGYLSLAMVPELPFPWLKDDIQAQAPRLGAYVKKLGGRCFGGSVDAAAVLSG IQAGTGSRLPWQIPERISLGGIGLRIFEGIADSIPVVKDIRLSRRLKQMGQDKSLESEEV LALADGYKREALTSAATVALGLGMFMVYLFTAGPLQVSFGTEDDHSSSSAENEEAGGDDG EEKGEKGSSGKPFDLGEVGSMLGL
